# Deep Learning of the Retina Enables Phenome- and Genome-Wide Analyses of the Microvasculature

**DOI:** 10.1161/CIRCULATIONAHA.121.057709

**Published:** 2021-11-08

**Authors:** Seyedeh Maryam Zekavat, Vineet K. Raghu, Mark Trinder, Yixuan Ye, Satoshi Koyama, Michael C. Honigberg, Zhi Yu, Akhil Pampana, Sarah Urbut, Sara Haidermota, Declan P. O’Regan, Hongyu Zhao, Patrick T. Ellinor, Ayellet V. Segrè, Tobias Elze, Janey L. Wiggs, James Martone, Ron A. Adelman, Nazlee Zebardast, Lucian Del Priore, Jay C. Wang, Pradeep Natarajan

**Affiliations:** Department of Ophthalmology and Visual Science, Yale School of Medicine, New Haven, CT (S.M.Z., J.M., R.A.A., L.D.P., J.C.W.).; Computational Biology & Bioinformatics Program (S.M.Z., Y.Y., H.Z.), Yale University, New Haven, CT.; School of Public Health (H.Z.), Yale University, New Haven, CT.; Program in Medical and Population Genetics and Cardiovascular Disease Initiative, Broad Institute of MIT and Harvard, Cambridge, MA (S.M.Z., V.K.R., M.T., S.K., M.C.H., Z.Y., A.P., S.U., P.T.E., P.N.).; Cardiovascular Research Center (S.M.Z., V.K.R., M.C.H., S.U., S.H., P.T.E., P.N.), Massachusetts General Hospital, Harvard Medical School, Boston.; Cardiovascular Imaging Research Center (V.K.R.), Massachusetts General Hospital, Harvard Medical School, Boston.; Centre for Heart Lung Innovation, University of British Columbia, Vancouver, Canada (M.T.).; MRC London Institute of Medical Sciences, Imperial College London, UK (D.P.O.).; Department of Ophthalmology, Massachusetts Eye and Ear, Harvard Medical School, Boston (A.V.S., T.E., J.L.W., N.Z.).

**Keywords:** deep learning, epidemiology, genomics, mendelian randomization analysis, microvessels, retina

## Abstract

**Background:**

The microvasculature, the smallest blood vessels in the body, has key roles in maintenance of organ health and tumorigenesis. The retinal fundus is a window for human in vivo noninvasive assessment of the microvasculature. Large-scale complementary machine learning-based assessment of the retinal vasculature with phenome-wide and genome-wide analyses may yield new insights into human health and disease.

**Methods:**

We used 97 895 retinal fundus images from 54 813 UK Biobank participants. Using convolutional neural networks to segment the retinal microvasculature, we calculated vascular density and fractal dimension as a measure of vascular branching complexity. We associated these indices with 1866 incident *International Classification of Diseases*–based conditions (median 10-year follow-up) and 88 quantitative traits, adjusting for age, sex, smoking status, and ethnicity.

**Results:**

Low retinal vascular fractal dimension and density were significantly associated with higher risks for incident mortality, hypertension, congestive heart failure, renal failure, type 2 diabetes, sleep apnea, anemia, and multiple ocular conditions, as well as corresponding quantitative traits. Genome-wide association of vascular fractal dimension and density identified 7 and 13 novel loci, respectively, that were enriched for pathways linked to angiogenesis (eg, vascular endothelial growth factor, platelet-derived growth factor receptor, angiopoietin, and WNT signaling pathways) and inflammation (eg, interleukin, cytokine signaling).

**Conclusions:**

Our results indicate that the retinal vasculature may serve as a biomarker for future cardiometabolic and ocular disease and provide insights into genes and biological pathways influencing microvascular indices. Moreover, such a framework highlights how deep learning of images can quantify an interpretable phenotype for integration with electronic health record, biomarker, and genetic data to inform risk prediction and risk modification.

## Abstract 2

Supplemental Digital Content is available in the text.

**Clinical Perspective**

**What Is New?**

- We leveraged deep learning to quantify geometric microvasculature indices across >100 000 retinal fundus photographs and used these indices toward characterizing phenome-wide clinical associations and genomic risk factors.
- Epidemiologically, low microvascular density and fractal dimension (a measure of vascular branching complexity) were associated with higher risk of future mortality and cardiometabolic and ocular disease; genetically, these microvascular indices were enriched in pathways related to angiogenesis and inflammation.
- Genetically higher risk for hypertension and diabetes was associated with lower microvascular density; in turn, genetically lower microvascular density was independently associated with higher risk of retinal detachments and skin cancer.

**What Are the Clinical Implications?**

- We illustrate the potential for deep learning of the retina to understand the microvasculature among humans in vivo, with wide applications across diseases.
- Retinal microvascular indices may be clinically useful as biomarkers of cardiometabolic disease severity and for risk prediction of ocular conditions; however, more research is required to assess clinical efficacy.
- Genetic contributors to microvascular indices, including those influencing angiogenesis and inflammation, may provide insight into therapeutic targets for microvascular disease in the eye, cancer, and diseases in other tissues.

The microvasculature influences health and disease throughout all organ systems. Dysregulation of the microvasculature contributes to many ocular and systemic conditions.^1–5^ The formation of blood vessels is controlled by 2 processes: vasculogenesis and angiogenesis. Vasculogenesis involves the differentiation of endothelial cells from mesodermal precursors and is under control of many signaling cues, including the WNT/B-catenin^6^ and Notch^7^ signaling pathways. Angiogenesis then follows a series of sequential steps for vascular branching and is mediated by growth factors and cytokines, including vascular endothelial growth factor (VEGF), fibroblast growth factor, tumor necrosis factor-α, transforming growth factor-β, platelet-derived growth factor, and angiopoietins, as well as intracellular signaling pathways incorporating Rho GTPases, protein kinase C, and Notch signaling.^8^ The microvasculature is also of significance in cancer, wherein angiogenesis is necessary for tumor growth and enables metastasis.^9^ Antiangiogenic agents such as anti-VEGF antibodies are key aspects of cancer therapy, whereby the actions of VEGF, which is induced by the tumor microenvironment (eg, hypoxia) and stimulates abnormal neovascularization, are inhibited.^9^ The same anti-VEGF therapies that treat cancers are also a mainstay of treatment for neovascularization and macular edema secondary to neovascular age-related macular degeneration and proliferative diabetic retinopathy, respectively.^10,11^

Given the anatomic and physiological similarities between the retinal microvasculature and that of other organs, fundus photographs of the retina allow noninvasive in vivo assessment of the microvasculature. Current computerized approaches of assessing the microvasculature include retinal vessel caliber estimation of arterioles and venules and their ratio.^12^ Smaller studies have shown that smaller retinal vascular caliber and smaller arteriolar/venular ratio are linked to antecedent and future hypertension.^13–17^ Furthermore, retinal vascular caliber is associated with incident stroke.^18–20^ However, associations with coronary artery disease and renal disease are less consistent.^21–23^ Current approaches are now able to extract geometric and branching patterns of the retinal microvasculature such as retinal vascular tortuosity,^24^ fractal dimension (FD),^25–31^ and vascular density.^32–36^ Such indices are more consistently linked to stroke, coronary artery disease, and renal disease in small cross-sectional analyses of ≈1000 individuals.^37-41^ The extent to which retinal microvasculature geometric alterations may be linked to incident phenome-wide consequences is poorly understood. In addition, complementary genetic discovery analyses of the retinal microvasculature offer the prospects of identifying new therapeutic targets for both ocular and nonocular conditions.

Here, we leveraged deep learning for automated image quality control and segmentation of the microvasculature across >100 000 retinal fundus photographs. We subsequently quantified 2 vascular features: branching complexity as measured with FD^42^ and vascular density, defined as the total number of segmented pixels given a consistent field of view and fixed pixel dimensions across all individuals. We then performed a phenome-wide association study (PheWAS) for FD and vascular density across 1866 phenotypes and 88 quantitative clinical traits and biomarkers. Secondarily, we performed genome-wide association studies (GWASs) for FD and vascular density across common variants in the genome, as well as a rare variant association study across rare predicted disruptive variants in the genome. We leveraged these results to investigate causal relationships through mendelian randomization. Overall, our study highlights how deep learning enables a large-scale, unbiased connection between the retinal microvasculature and clinical outcomes (Figure 1A).

**Figure 1.**
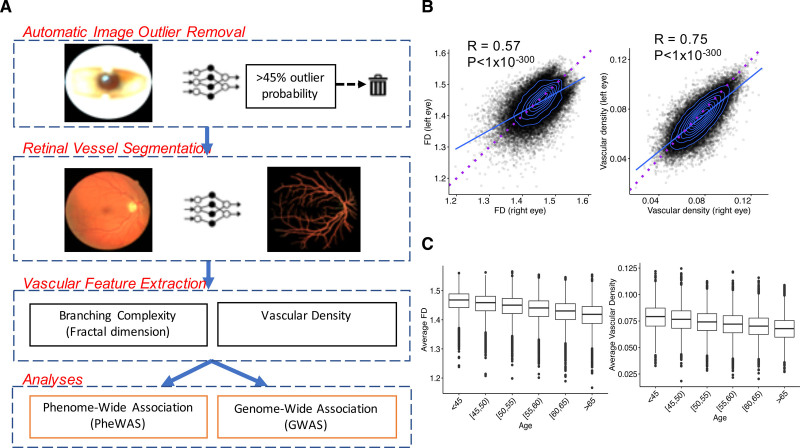
**Study schematic and vascular features. A**, Here, we first used deep learning toward large-scale automated removal of low-quality images, followed by vessel segmentation. Next, using the vascular segmentations, we quantified 2 vascular indices: branching complexity as measured by fractal dimension (FD) and vascular density. Last, phenome- and genome-wide association analyses of retinal FD and vascular density were performed to discover phenotypes associated with the microvasculature and genotypes influencing these vascular indices. **B**, Significant Spearman correlations were observed between FD and vascular density of the right vs left eyes, with right eyes having significantly higher FD and vascular density compared with left eyes. Blue line reflects best-fit line; dotted purple line reflects the unity line (*x*=*y*). **C**, Relationship of FD and vascular density (averaged across right and left eyes) with age.

## Methods

### UK Biobank Cohort, Retinal Fundus Imaging, and Quality Control

The UK Biobank is a population-based cohort of ≈500 000 participants recruited from 2006 to 2010 with existing genomic and longitudinal phenotypic data and median 10-year follow-up.^43^ Baseline assessments were conducted at 22 assessment centers across the United Kingdom with sample collections, including blood-derived DNA. Retinal fundus imaging was performed at enrollment with the TOPCON 3D OCT 1000 Mk2 instrument, which takes a 3-dimensional scan and photograph of the retina with a magnified photograph of the fundus. Of the 67 339 genotyped individuals with retinal imaging available at enrollment, we analyzed 97 895 images across 54 813 participants after applying the quality control filters as indicated below. Use of the data was approved by the Massachusetts General Hospital Institutional Review Board (protocol 2013P001840) and facilitated through UK Biobank Application 7089. Details on UK Biobank array genotyping,^43^ whole-exome sequencing,^44,45^ genomic quality control,^46–48^ sample exclusion criteria, and poor-quality fundus image removal^49,50^ are provided in the Supplemental Methods.

### Deep Learning for Vessel Segmentation

An overview of previous methods for retinal vessel segmentation^42,51–56^ is provided in the Supplemental Methods. Here, we developed a deep learning ensemble model of U-Nets^57^ on the Google Cloud’s artificial intelligence platform to automatically segment vasculature from retinal fundus photographs. The model was developed from 90 photographs and associated hand-drawn segmentations from 3 publicly available data sets: (1) Digital Retinal Images for Vessel Extraction, photographs from a diabetic retinopathy screening program in the Netherlands of subjects 25 to 90 years of age; (2) fundus photographs with hand-labeled vessel segmentations from the Structured Analysis of the Retina database^58^ extracted from clinical visits to the Shiley Eye Center at the University of California, San Diego; and (3) CHASE (Child Heart and Health Study in England),^59^ retinal fundus photographs of 9- and 10-year-old children of different ethnic origin from England. Seventy-five images were used for training and hyperparameter tuning of the model, and the remaining 15 images were used as an independent validation data set (with the 3 data sets proportionally represented). A second external validation data set consisted of 143 images from the Automated Retinal Image Analysis database. These were retinal fundus photos from adults with age-related macular degeneration, adults with diabetic retinopathy, and healthy control subjects collected between 2004 and 2006 from the St. Paul’s Eye Unit in Liverpool, UK.^60^ Details on model training and FD^42,61^ and vascular density calculation are provided in the Supplemental Methods.

### PheWAS Analyses

Four sets of PheWAS analyses were performed, corresponding to association of retinal vascular FD and vascular density with (1) prevalent phenotypes at enrollment,^62,63^ (2) incident phenotypes developed after enrollment,^62,63^ (3) quantitative systemic biomarkers, and (4) quantitative ocular traits,^46–48^ as detailed in the Supplemental Methods including the statistical analysis. All models were adjusted for age, age squared, sex, smoking status (current/previous/never smoker), and ethnicity (data field 21000). For each analysis, statistical significance was defined with false discovery rate–corrected *P*<0.05. Sensitivity analyses additionally adjusting for hypertension, type 2 diabetes, myopia, and spherical equivalence^64^ were performed.

### Genomic Analyses

GWASs were performed with Hail-0.2 software on the Google Cloud among individuals with retinal fundus imaging at enrollment, as done previously. A linear regression model was used for analysis, with adjustment for age, age squared, sex, ever smoking, the first 10 principal components of genetic ancestry, and genotyping array. Secondary in silico analyses,^65–67^ polygenic risk score (PRS) PheWAS analyses, mendelian randomization,^47,68^ and rare variant burden analyses^69–71^ are detailed in the Supplemental Methods, including the statistical analysis. The full retinal vascular FD and density GWAS summary statistics are available through the Common Metabolic Disease Knowledge Portal (https://hugeamp.org/downloads.html).

### Code Availability

Deep learning methods for vessel segmentation and quantification are freely available through GitHub (https://github.com/vineet1992/Retina-Seg). Code for all other computations is available on reasonable request to the corresponding authors.

## Results

### Deep Learning for Automated Image Quality Control and Vessel Segmentation

We first performed automated image outlier detection by developing a convolutional neural network trained on a sample of 794 retinal fundus photographs from the UK Biobank. This model achieved a sensitivity of 97.4% and a specificity of 100.0% for detecting poor-quality images in the independent testing set of 206 fundus photographs. We then applied this model across 134 653 photographs acquired at the UK Biobank enrollment visit across 67 339 individuals. This resulted in removal of 26% of the original images with 99 736 images from 55 603 participants remaining, similar to filtering rates from other studies.^49,50^ Further details on sensitivity analyses for poor-quality image removal are provided in the Supplemental Methods (Table S1).

To implement large-scale vessel segmentation, we developed an ensemble of deep convolutional neural networks as detailed in the Methods section. On the 15-image testing data set, this ensemble model achieved an 82.1% Dice similarity coefficient (a measure of spatial overlap accuracy), 97.4% pixel-wise accuracy, 99.1% area under the curve, and correlation of 0.92 for FD and 0.88 for vascular density with the true hand-labeled vessel segmentations on the independent testing data set (Figure S1). These results were comparable or superior to results of other deep learning-based approaches.^52^ On the Automated Retinal Image Analysis external validation data set, the model achieved an accuracy score of 95.6% and an area under the curve of 97.4% higher than state-of-the-art image processing–based approaches (Figure S2).^51,53–56,72,73^ Although our model had a lower Dice coefficient in the Automated Retinal Image Analysis data set, (71.1%), FD and vessel density correlations remained high (ie, 0.93 and 0.82, respectively). Further sample quality control filters were implemented for analysis to filter to individuals consenting to genetic analysis with genotypic-phenotypic sex concordance, resulting in a total of 97 895 images across 54 813 participants used in the analyses.

### Baseline Characteristics

Baseline characteristics across the 54 813 analyzed individuals (mean age, 56 years [SD 8 years]; female, 30 015 [55%]; ever smokers, 23 987 [44%]) are presented in Table S2 and are discussed in the Supplemental Results.

### Retinal Vascular Density and FD Quantification

The calculated retinal vascular density and FD, although highly correlated between right and left eyes (FD: *R*_Spearman_=0.57, *P*<1×10^−^^300^; density: *R*_Spearman_=0.75, *P*<1×10^−^^300^), were both consistently higher in right eyes compared with left eyes (paired *t* test for FD: mean difference, 0.0065, *P*=2×10^−^^264^; density: mean difference, 0.0031, *P*<1×10^−^^300^; Figure 1B). This relationship was independent of handedness and was maintained across right-handed, left-handed, and ambidextrous individuals (Figure S3). Retinal vascular density and FD across both eyes were strongly correlated (right eye: *R*_Spearman_=0.77, *P*<1×10^−^^300^; left eye: *R*_Spearman_=0.78, *P*<1×10^−300^). Univariable and multivariable associations with vascular FD and density (Figure 1C, Figure S4, and Tables S3 and S4) are discussed in the Supplemental Results.

### Phenome-Wide Association Study

**Figure 2.**
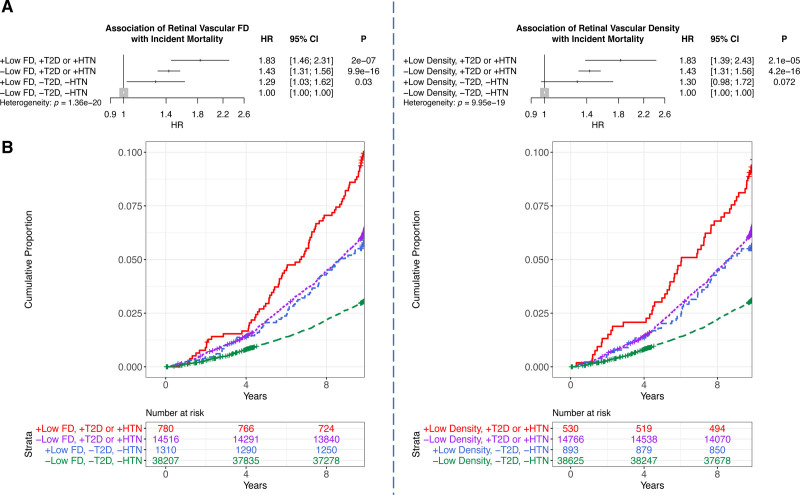
**Association of retinal vascular FD and density with incident mortality. A**, Association of low (≥2 SD below the mean) fractal dimension (FD) and density with incident mortality, stratified by whether the person has prevalent type 2 diabetes (T2D) or hypertension (HTN) at time of image acquisition. Analyses are adjusted for age, age squared, sex, smoking status, and ethnicity. **B**, Cumulative incidence of mortality across individuals with low FD and density who have a diagnosis of prevalent T2D or hypertension compared with those who do not. HR indicates hazard ratio.

We first associated retinal vascular FD and density with incident mortality, identifying that the incidence of mortality is strongest among individuals with low FD and density who have either type 2 diabetes or hypertension (Figure 2). Low FD, defined as 2 SDs below the mean, was more strongly associated with incident mortality among those with prevalent type 2 diabetes or hypertension (hazard ratio [HR], 1.83 [95% CI, 1.46–2.3]; *P*=2.01×10^−^^7^) than those without either condition (HR, 1.29 [95% CI, 1.03–1.62]; *P*=0.03, *P*_heterogeneity_=1.36×10^−^^20^) after adjustment for age, age squared, sex, smoking status, and ethnicity. Similarly, a 2-SD lower vascular density was associated with incident mortality (HR, 1.83 [95% CI, 1.39–2.42]; *P*=2.1×10^−^^5^) among those with prevalent type 2 diabetes or hypertension, but no significant association was detected among those without either condition (HR, 1.30 [95% CI, 0.976–1.72]; *P*=0.072; *P*_heterogeneity_=9.95×10^−^^19^) adjusted for age, age squared, sex, smoking status, and ethnicity (Figure 2).

**Figure 3.**
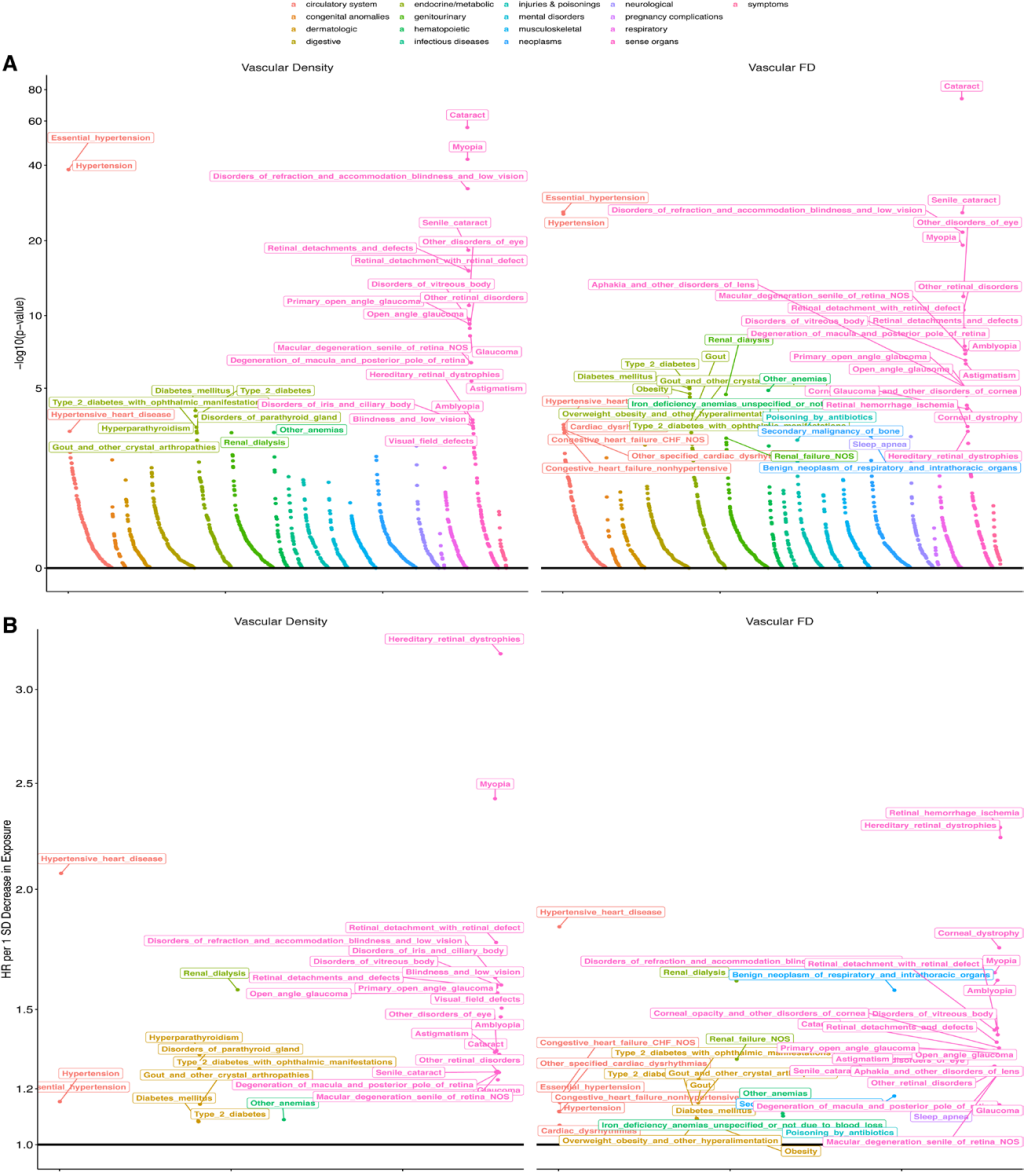
**Phenome-wide associations with incident disease. A**, –Log10(*P* value) of associations of retinal vascular density and fractal dimension (FD) with incident disease plotted as grouped by phenotypic category. Associations were performed with Cox proportional hazards models adjusted for age, age squared, sex, smoking status, and ethnicity. **B**, Hazard ratio (HR) per 1-SD decrease in either vascular density (**left**) or FD (**right**). Labeled phenotypes across both plots have false discovery rate–corrected *P*<0.05. The *x* axis reflects an organized grouping of the phenotypes by phenotypic category and *P* value of association.

Next, phenome-wide association was performed across all 1866 hierarchical phenotypes (ie, phecodes) defined from the Phecode Map 1.2 *International Classification of Diseases-9* and *-10* groupings^62^ (Supplemental Methods). Analysis was performed separately between retinal vascular FD and density across all prevalent phecodes diagnosed before fundus image acquisition (Figure S5 and Table S5), incident phecodes first diagnosed after fundus image acquisition (Figure 3 and Table S6), and quantitative clinical traits and serological biomarkers ascertained at fundus image acquisition (Figure S6 and Table S7). In all models, we adjusted for age, age squared, sex, smoking status, and ethnicity. Across all these analyses, significant associations were identified between the retinal vasculature and both systemic and ocular conditions.

With respect to associations with systemic conditions, we found that low vascular density and FD were significantly (false discovery rate–corrected *P*<0.05) associated with higher odds of prevalent and incident cardiometabolic phenotypes, including hypertension, diabetes, anemia, and pulmonary disease.

First, each 1-SD decrease in retinal vascular density and FD was associated with higher odds of having a history of hypertension (odds ratio [OR]_Density_, 1.08; OR_FD_, 1.09) and higher risk of developing new-onset hypertension (HR_Density_, 1.15; HR_FD_, 1.12), with strongest incident risk linked to new-onset hypertensive heart disease (HR_Density_, 2.07; HR_FD_, 1.84). Accordingly, systolic blood pressure (SBP) and diastolic blood pressure (DBP) were associated with lower retinal vascular density (β_SBP_, −0.13; β_DBP_, −0.12) and FD (β_SBP_, −0.10; β_DBP_, −0.08; in units of SD in retinal vascular trait per 1-SD increase in blood pressure). In secondary analyses, retinal vascular density and FD predicted incident hypertension even after adjustment for quantitative SBP and DBP (Figure S7).

Second, lower retinal vascular density and FD were associated with higher odds of having a history of diabetes, diabetic retinopathy, and ophthalmic manifestations of type 1 and type 2 diabetes, with the strongest effect estimate for prevalent (OR_Density_, 3.38; OR_FD_, 3.25 per SD) and incident (HR_Density_, 1.56; HR_FD_, 1.42) ophthalmic manifestations of type 1 diabetes. However, additional adjustment of the incident type 2 diabetes association by hemoglobin A1c (HbA1c) at enrollment rendered the associations no longer statistically significant (Figure S8). Accordingly, each 1-SD increase in glucose and HbA1c was associated with lower retinal vascular density (β_Glucose_, −0.03; β_HbA1c_, −0.01) and FD (β_Glucose_, −0.02; β_HbA1c_, −0.02; in units of SD in retinal vascular trait per 1-SD increase in blood pressure). Other associations with traits linked to metabolic disease were also detected, for example, body mass index and body fat percent with lower vascular density and FD. Associations with pulmonary phenotypes were also identified; lower retinal FD was associated with pulmonary function (forced expiratory volume in the first second of expiration and forced vital capacity) and sleep apnea (HR_FD_, 1.13).

Third, a strong link between anemia and lower retinal vasculature density and FD was also observed, with each 1-SD decrease in retinal vascular density and FD being associated with higher odds of having a history of iron-deficiency anemia (OR_Density_, 1.19; OR_FD_, 1.24). Accordingly, each 1-SD decrease in hematocrit percentage, hemoglobin concentration, and red blood cell count was associated with lower retinal vascular density and FD.

Multiple associations were also identified with ocular conditions, including associations across both anterior and posterior segments of the eye. Retinal vascular density and FD also were associated with incident ocular diagnoses, including conditions influencing both the posterior segment—myopia (HR_Density_, 2.42; HR_FD_, 1.65), age-related macular degeneration of the retina (HR_Density_, 1.26; HR_FD_, 1.25), retinal detachments with defects (HR_Density_, 1.77; HR_FD_, 1.43)—and the anterior segment—primary open-angle glaucoma (HR_Density_, 1.56; HR_FD_, 1.33), cataracts (HR_Density_, 1.34; HR_FD_, 1.34; Figure 3 and Table S6). Lower retinal FD and density were also associated with higher intraocular pressure and reduced visual acuity (Figure S9 and Table S5).

Further sensitivity analyses adjusted for prevalent hypertension and diabetes (Figure S10 and Table S8) and mean spherical equivalent (Figure S11 and Table S9) are discussed in the Supplemental Results.

### Common Variant GWAS and In Silico Analyses

**Figure 4.**
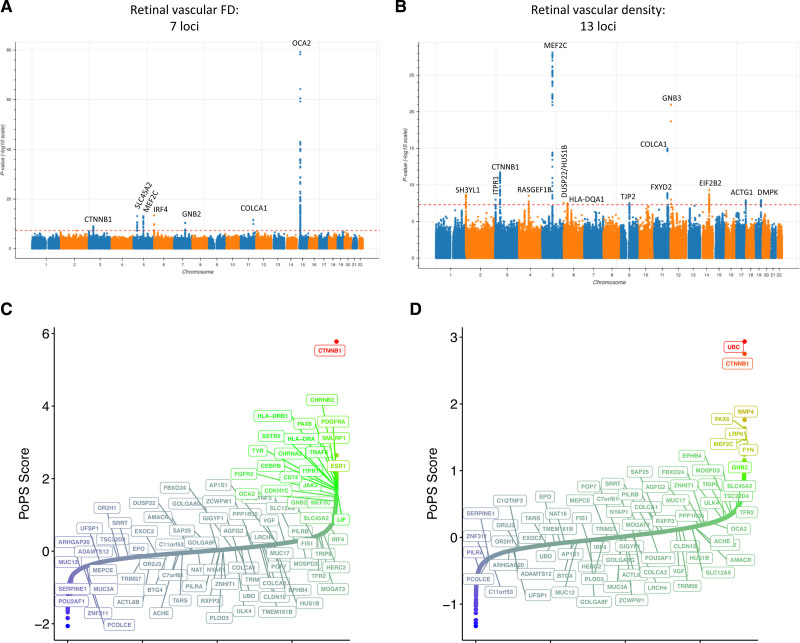
**Genome-wide association studies and gene prioritization. A** and **B**, Manhattan plots visualizing the genome-wide association results for retinal vascular fractal dimension (FD; **A**) and density (**B**), which identify 7 and 13 loci, respectively. Gene prioritization with the Polygenic Priority Score (PoPS) method.^67^ **C** and **D**, Prioritized genes at each locus (see locus-specific prioritizations in Figure S15). The locus-specific genes prioritized by PoPS at each locus are labeled in **A** and **B**. The *x* axes in **C** and **D** are arbitrary and reflect the ordered genes by PoPS score.

GWASs for retinal vascular density and FD were carried out across 38 932 unrelated individuals and 15 580 782 variants with minor allele frequency >0.001 in the UK Biobank, identifying 13 and 7 genome-wide–significant (*P*<5×10^−^^8^) loci, respectively (Figure 4A and 4B and Tables S10 and S11). Genomic heritability was estimated at 14.1% (SE, 1.6%) for FD and 21.0% (SE, 1.8%) for vascular density. Significant correlation was observed between prioritized genome-wide–significant, independent variants identified through fine mapping^66^ from the retinal vascular density and FD GWASs (Figure S12 and Table S12), with all loci from the vascular FD GWASs showing suggestive significance in the vascular density GWASs, and only 1 locus from the vascular density GWASs not showing significance in the FD GWASs (*TJP2* intron variant, rs56207218-C; allele frequency, 21.2%; β_Density_, −0.046 SD, *P*_Density_=3.01×10^−^^8^; β_FD_, −0.016, *P*_FD_=0.053). A missense variant (rs5442-A, p.Gly272Ser; allele frequency, 7.1%) in *GNB3*, encoding the G-protein β polypeptide 3 protein, was the top variant associated with vascular density in chromosome 12 (β, −0.13 SD; *P*=1.11×10^−21^) and is predicted to be deleterious by several in silico prediction tools, including PolyPhen,^74^ SIFT,^75^ and PrimateAI.^76^ It was also suggestively associated with vascular FD (β, −0.069 SD; *P*=1.06×10^−^^7^). Further sensitivity analyses and comparison with variants identified in a previous GWAS of vascular tortuosity^24^ are provided in the Supplemental Results (Figure S13 and S14 and Table S12).

After performing GWAS, we performed 3 sets of analyses to prioritize the genes, traits, and biological pathways linked to the GWAS loci.

First, we performed causal gene prioritization using Polygenic Priority Score (PoPS), which combines information across gene expression data from 73 publicly available bulk RNA sequencing and scRNA sequencing databases (including from retinal tissues), 8479 biological pathways, and 8718 protein-protein interactions to prioritize likely implicated genes at each GWAS locus using GWAS summary statistics^67^ (Figure 4C and 4D and Figure S15). Across the majority of top loci, PoPS prioritized 1 gene (Table S13 and Figure S15). The top prioritized genes at the 7 FD loci include *CTNNB1*, *OCA2*, *SLC45A2*, *MEF2C*, *IRF4*, and *GNB2*, with similar PoPS identified for *COLCA2* and *COLCA1* at the associated chromosome 11 locus. Several of the prioritized PoPS genes at the 13 vascular density loci overlapped with those from the FD GWAS, including at the following PoPS-prioritized genes: *COLCA1/COLCA2* locus, *CTNNB1, MEF2C*, and *IRF4* (which was prioritized as *DUSP22/HUS1B* in the density GWAS). Additional PoPS-prioritized genes uniquely genome-wide significant in the vascular density GWAS included *FXYD2*, *GNB3*, *EIF2B2*, *ACTG1*, *DMPK*, *SH3YL1*, *ITPR1*, *RASGEF1B*, *HLA-DA1*, and *TJP2*. Outside of these loci, several other genes were also suggestively prioritized by PoPS, including *HLA-DRA*, *HLA-DRB1*, *ESR1*, *FGFR2*, *PDGFRA*, and *TYR* for FD and *UBC* for vascular density (Figure S15).

Second, we identified other traits associated with the top variants, assessing traits with significant associations from the PheWAS (such as systemic traits including blood pressure and diabetes, as well as ocular traits such as retinal detachment, myopia, glaucoma, diabetic retinopathy, and macular degeneration), in addition to traits showing strong associations on PhenoScanner for top variants (including melanoma, eye cancer, and skin color). Genetic correlation analyses across the genome identified significant inverse correlations between retinal vascular FD and density with previous published loci for myopia, age-related cataracts, lighter skin color, retinal detachment, SBP, and DBP (Figure S16a and S16b and Table S14). Further fine mapping of genome-wide–significant loci was performed to prioritize potentially causal variants^66^ (Table S15). Retinal vascular density– and FD-lowering alleles at the fine-mapped variants had heterogeneous effects on the previous phenotypes assessed in genetic correlation analyses (Figure S16c and S16d and Table S16). In particular, retinal density– and FD-lowering alleles at several PoPS-prioritized genes showed associations with higher risk of skin neoplasms, malignant melanoma, and eye cancer (*IRF4/DUSP22*, *SLC45A2*); a separate set of loci showed associations with lighter skin color (*GNB2*, *ACTG1*). One locus showing consistency with the phenotypic associations previously described was the retinal vascular density–lowering variant rs8070929-T (β_Density_, −0.04 SD; *P*_Density_=1.27×10^−8^) at the PoPS-prioritized gene *ACTG1* encoding the actin gamma 1 protein, for which each retinal vascular density-–lowering allele was genome-wide significantly associated with higher risk of advanced age-related macular degeneration (OR, 1.13; *P*=1.65×10^−^^11^), reduced refractive error (β=−0.03; *P*=2.68×10^−^^30^), and lighter skin color (β=0.01; *P*=8.17×10^−^^27^) and suggestively associated with higher risk of retinal detachment, age-related cataracts, skin neoplasms, and higher DBP (*P*<0.05; Figure S16c and S16d and Table S16).

**Figure 5.**
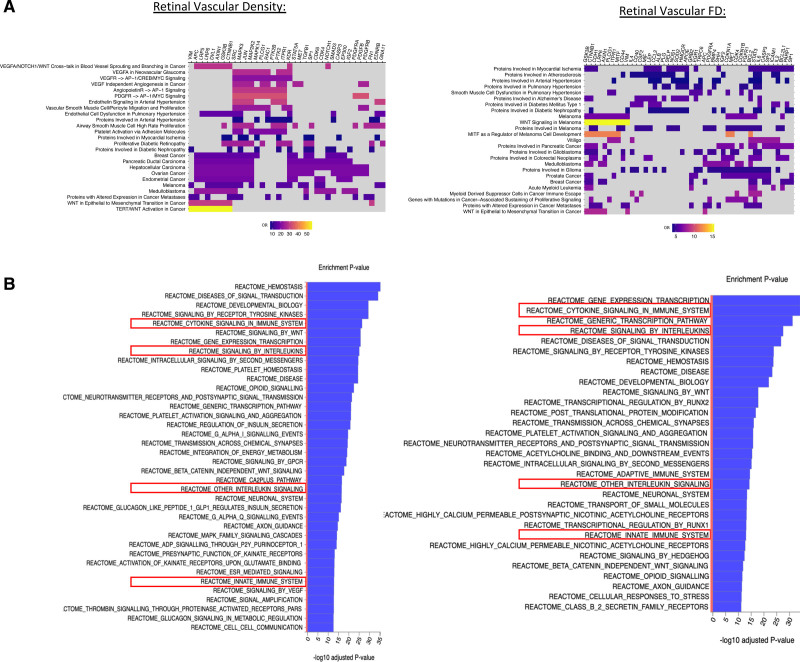
**Pathway enrichment analysis.** Pathway enrichment analyses of the retinal vascular fractal dimension (FD) and density genome-wide association study results were performed using the prioritized genes with Polygenic Priority Score *z* score >1 across the (**A**) Elsevier pathways and (**B**) Reactome pathways. Top Bonferroni-significant results are listed (**A**), along with the enrichment odds ratios (ORs) and the enrichment *P* values (**B**). Further details on genes included in each pathway and enrichment statistics are given in Tables S17 and S18.

Third, we performed gene set enrichment analyses to identify biological pathways overrepresented by the prioritized genes. Using the list of prioritized genes with PoPS *z* scores >1, we performed enrichment analyses across Elsevier pathways,^77^ identifying Bonferroni-significant enrichment for VEGF/VEGFA/VEGF receptor/platelet-derived growth factor receptor/angiopoietin signaling, WNT signaling, endothelin signaling, atherosclerosis, arterial and pulmonary hypertension, melanoma, and proteins with altered expression in cancers (Figure 5A and 5B). Pathway enrichment analysis using the Reactome pathways^78^ additionally identified an enrichment of inflammatory pathways, including interleukin and chemokine pathways for PoPS genes prioritized in both the retinal vascular density and FD GWASs (Figure 5C and 5D and Tables S17 and S18).

Further results from rare variant association study grouping together rare (minor allele frequency <1%), disruptive (ie, MetaSVM^70^ missense deleterious or high-confidence loss-of-function variants^71^) variants by gene are discussed in the Supplemental Results (Figure S17 and Tables S19 and S20).

### One-Sample Mendelian Randomization

Here, 1-sample Mendelian randomization was performed to assess the putative causal relationships between systemic phenotypes on the retinal microvasculature and the retinal microvasculature on disease, specifically on ocular phenotypes.

**Figure 6.**
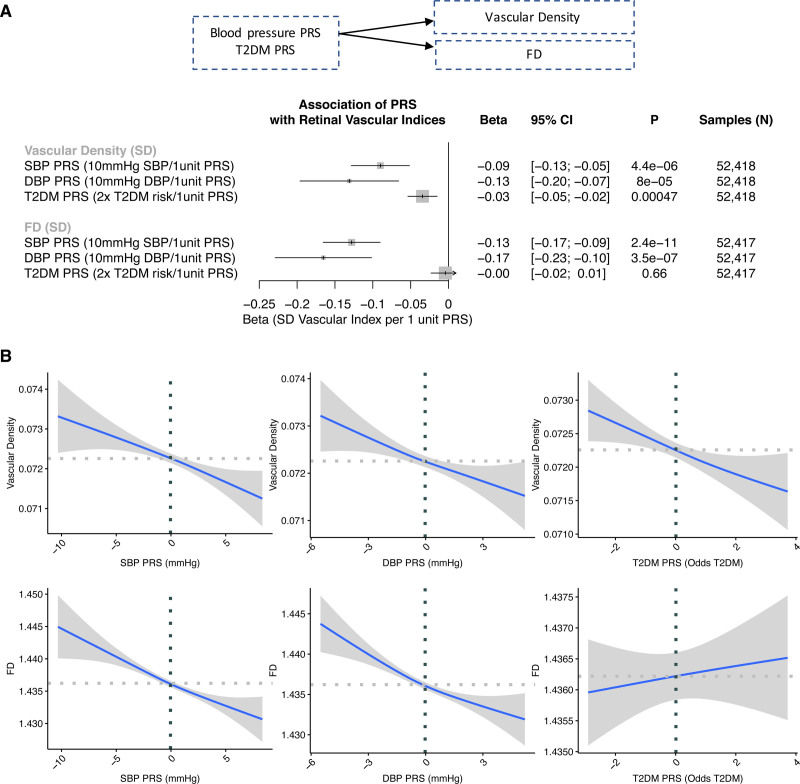
**One-sample mendelian randomization for DBP PRS, SBP PRS, and T2D PRS on retinal vascular density and FD. A**, Association of the diastolic blood pressure (DBP), systolic blood pressure (SBP), and type 2 diabetes (T2D) polygenic risk score (PRS) with normalized retinal vascular density and fractal dimension (FD) in a linear regression model adjusted for age, age squared, sex, smoking status, and the first 10 principal components of genetic ancestry. **B**, Relationship of the SBP, DBP, and T2D PRSs with retinal vascular FD and density. Horizontal and vertical dotted lines reflect the average value for the respective axis. Shaded gray region reflects the 95% CI using a restricted maximum likelihood generalized additive model with integrated smoothness from the gam() function in R.

First, given the strong link between hypertension and diabetes and retinal vascular indices in the PheWAS, 1-sample mendelian randomization was performed using PRSs for SBP (75 variants), DBP (75 variants), and type 2 diabetes (64 variants) comprising genome-wide–significant (*P*<5×10^−^^8^), independent variants from European GWASs external to the UK Biobank, as done previously.^47,68^ These PRSs were then associated with retinal vascular density and FD adjusted for age, age squared, sex, smoking status, and European ancestry. Significant associations were identified between the blood pressure PRS and both vascular density and FD, with the direction of association matching that identified previously in the PheWAS, with each 10 mm Hg in genetically elevated SBP being associated with a 0.09-SD decrease in vascular density (*P*=4.4×10^−^^6^) and 0.13-SD decrease in FD (*P*=2.4×10^−^^11^), and each 10 mm Hg in genetically elevated DBP being associated with a 0.13-SD decrease in vascular density (*P*=8.0×10^−^^5^) and 0.17-SD decrease in FD (*P*=3.5×10^−^^7^; Figure 6). Each 2-fold higher genetic risk for type 2 diabetes was associated with a 0.03-SD decrease in retinal vascular density (*P*=4.7×10^−^^4^); no significant association was observed between the type 2 diabetes PRS and FD.

Second, to assess the phenome-wide influence of genetically elevated microvascular indices, PRSs for FD and vascular density were developed consisting of independent, genome-wide–significant (*P*<5×10^−^^8^) variants from the GWAS and then assessed among an independent set of UK Biobank participants who were not included in the original GWAS study. The strongest association was identified between both the vascular density and FD PRSs and skin cancers (OR, 1.14 per 1-SD decrease in FD PRS, *P*=3.09×10^−^^43^; OR, 1.05 per 1-SD decrease in density PRS, *P*=1.7×10^−^^11^; Table S21 and Figure S18); these associations were largely robust to adjustment for self-reported skin color, sunlight exposure, and sunlight sensitivity (OR, 1.04 per 1-SD decrease in FD PRS, *P*=4.22×10^−^^5^; OR, 1.04 per 1-SD decrease in density PRS, *P*=1.4×10^−^^4^; Table S22).

**Figure 7.**
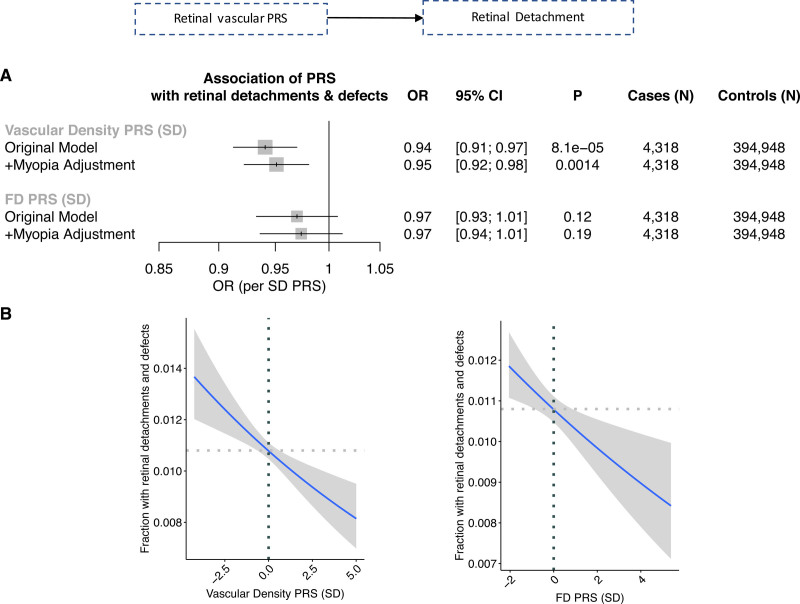
**One-sample mendelian randomization for vascular FD PRS and density PRS on retinal detachment. A**, Association of the vascular density PRS and fractal dimension (FD) polygenic risk score (PRS) with combined prevalent and incident retinal detachment. Original model includes the following covariates: age, age squared, sex, smoking status, and the first 10 principal components of genetic ancestry. +Myopia adjustment reflects additional adjustment for prevalent myopia at the time of image acquisition. **B**, Relationship of vascular density PRS and vascular FD PRS with fraction of individuals developing retinal detachments and defects during their lifetime. Horizontal and vertical dotted lines reflect the average value for the respective axis. Shaded gray region reflects the 95% CI using a restricted maximum likelihood binomial generalized additive model with integrated smoothness from the gam() function in R. OR indicates odds ratio.

Among the epidemiological ocular associations identified previously in the PheWAS, concordant 1-sample mendelian randomization directional association was identified between each 1-SD increase in vascular density PRS and lower myopia (OR, 0.92; *P*=9.5×10^−^^6^; Figure S19), as well as retinal detachments and defects (OR, 0.94; *P*=5.7×10^−^^4^), that remained significant after adjustment for myopia (OR, 0.95,; *P*=0.0014; Figure 7).

## Discussion

**Figure 8.**
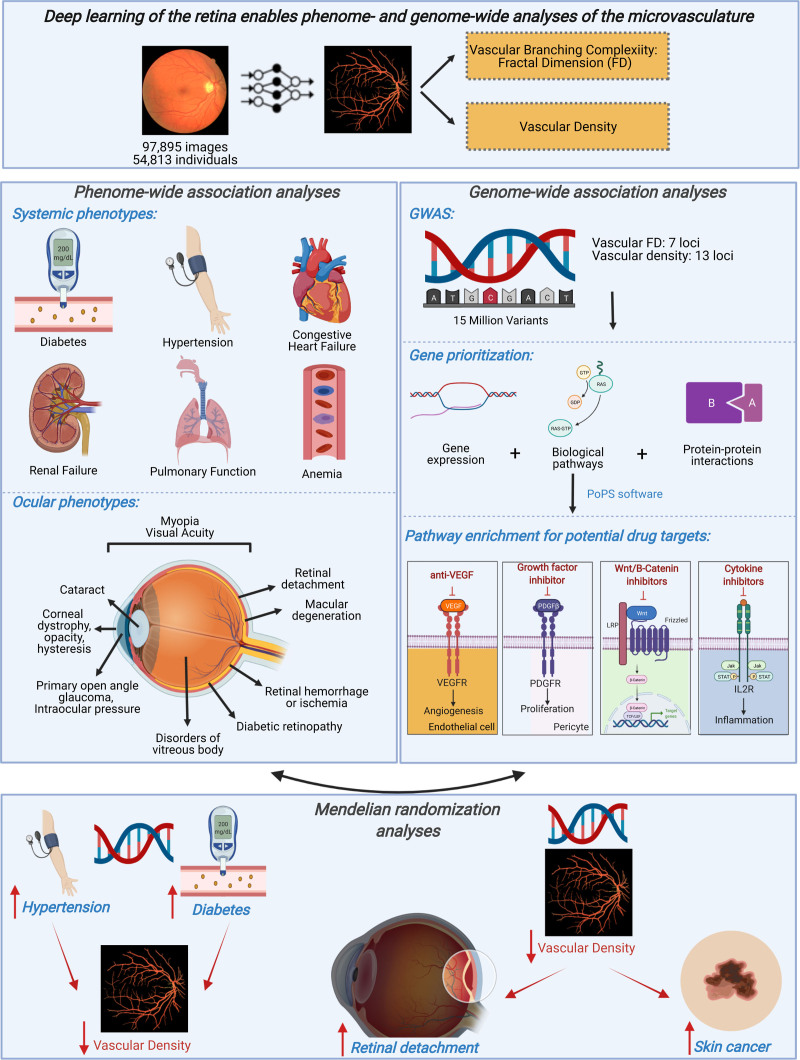
**Summary of key findings.** Here, we successfully implemented deep learning toward image quality control and vessel segmentation to extract 2 features of the retina: vascular density and fractal dimension (FD). Through phenome-wide analyses, we identified significant associations between low vascular density and FD and higher risk of multiple systemic and ocular phenotypes, including ocular conditions influencing both the anterior and posterior segments. Genome-wide association analyses of these microvascular indices discovered multiple loci enriched among pathways related to vascular biology, inflammation, and neovascularization in cancer and may hypothesize potential drug targets for risk modification. Mendelian randomization analyses identified that higher genetic risk for hypertension and type 2 diabetes is associated with lower microvascular density and that higher genetic risk for lower microvascular density is associated with retinal detachment (independently of myopia) and with skin cancer (independently of genetic ancestry principal components, self-reported skin color, and self-reported sun exposure and sun sensitivity). More broadly, our results illustrate the potential for using deep learning on retinal imaging to understand the microvasculature, with wide applications across diseases. This image was made with Biorender. GWAS indicates genome-wide association study.

In summary, we used deep learning to quantify in vivo microvascular architecture across nearly 100 000 human retinal fundus images and densely explore unbiased phenome-wide and genome-wide assessments (Figure 8). Through phenome-wide association analyses, we identified systemic and ocular phenotypes linked to the retinal microvasculature. Genome-wide association analyses identified 7 novel loci associated with microvascular branching complexity (as measured by FD) and 13 loci associated with vascular density, enriched for genes among pathways related to angiogenesis, cancer, and inflammation. Last, we used mendelian randomization to assess the causal relation between systemic conditions and microvascular indices, as well as between microvascular retinal indices and ocular conditions. Together, these findings permit several conclusions.

First, microvascular alterations detected from the retina are linked to diverse conditions, providing new insights into the microvasculature as a biomarker for systemic disease risk and severity. In particular, a lower microvascular density or fractal branching was associated with higher risk of incident mortality among those with prevalent hypertension or diabetes compared with those without either condition. This suggests that lower microvascular density and branching complexity are associated with higher severity of disease among those with existing cardiometabolic disease. We also observed significant associations between lower retinal vascular density and FD and higher prevalence of, and separately higher risk for, cardiometabolic (hypertension, hypertensive heart disease, diabetes, renal failure, elevated HbA1c, and body mass index), pulmonary (sleep apnea, abnormal pulmonary function tests), and hematopoietic (anemia) conditions. Retinal microvascular dysfunction may signify more widespread microvascular alterations. For example, hyperglycemia-induced cellular dysfunction and death are linked to widespread insufficient renewal of vascular endothelial and smooth muscle cells through sorbitol accumulation, glycosylation, and reactive oxygen formation.^26,79,80^ Furthermore, hypertension-induced sheer stress and anemia-induced deficits in gas and nutrient exchange may similarly impair the systemic microvasculature. Moreover, pulmonary conditions such as sleep apnea are a well-described clinical risk factor for ocular conditions.^81,82^ Of note, previous studies in small cohorts have been inconclusive or contradictory in the reported relationships between vascular FD and diabetes and hypertension.^25–28^

Second, we identify associations between lower retinal vascular density and FD and higher risk of future ocular conditions. Although previous studies in small cohorts have identified associations between retinal vascular indices and prevalent diabetic retinopathy,^25,26,37^ this is, to the best of our knowledge, one of the first large-scale studies identifying associations with diverse future ocular conditions. Specifically, we identify multiple associations between lower retinal vascular FD and density and higher risk of future incident conditions influencing the posterior segment of the eye (retinal detachment, diabetic retinopathy, macular degeneration, vitreous hemorrhage). In particular, retinal detachment is a potentially blinding ocular condition for which limited risk factors have been described, including ocular trauma, myopia, and family history. Here, we find evidence of significant association between lower retinal vascular density and FD and higher incidence of retinal detachment, independently of myopia. These findings suggest that identification of individuals with lower retinal vascular density may enable improved monitoring and blindness risk reduction in this high-risk population. Moreover, we also identify associations with the anterior segment of the eye (glaucoma, cataracts), suggesting that the retinal vasculature may have physiological significance beyond the retina and vitreous fluid. Our observations are aligned with previous studies hypothesizing a link between the retinal vasculature and normal-tension glaucoma.^83,84^ Hypothesized contributors to normal-tension glaucoma include vascular abnormalities impeding nutrient delivery to the inner retina, thereby resulting in ganglion cell degeneration.^83,84^ Together, these findings linking the retinal vasculature with ocular pathophysiology highlight the importance of the retinal microvasculature in ophthalmic health and help us understand the diverse mechanisms linking the retinal microvascular to impaired visual acuity and blindness.

Third, genome-wide association analyses identified genetic links between genes involved in angiogenesis, cancer, pigmentation, and inflammation and microvasculature architecture. We observed a significant enrichment in pathways related to angiogenesis (VEGF, platelet-derived growth factor, angiopoietin), which are currently therapeutically targeted to inhibit neovascularization in diabetic retinopathy, advanced age-related macular degeneration, and many cancers.^9–11^ A significant genetic correlation with skin color was also observed, and several of the fine-mapped top variants are strongly associated with skin neoplasms and lighter skin color (ie, at the PoPS-prioritized genes *IRF4*, *SLC45A2*, *OCA2*, *DUSP22*, *ACTG1*). Indeed, *OCA2* encodes the oculocutaneous albinism 2 protein that is known to result in lighter skin color and predisposes to skin cancers.^85^ Additionally identified in both the rare variant association study and GWASs were predicted deleterious variants in *MITF*, a transcription factor necessary for normal melanocyte differentiation. *MITF* has been associated with Waardenburg syndrome, characterized by pigmentation anomalies of eyes, hair, and skin.^86–88^ Previous work has also identified that MITF (melanocyte-inducing transcription factor) protein labeling in human tumor samples is strong around the vessels.^89^ Moreover, 2 of the GWAS loci identified across the FD and vascular density GWASs, namely *GNB3* and *GNB2*, are G proteins and are known to be key moderators of chemokine signal transduction pathways. Notably, the missense variant rs5442-A in *GNB3* variant has previously been associated with retinal microvascular diameter,^90^ hypertension,^91^ refractive error,^92,93^ and advanced age-related macular degeneration.^94^ Inflammatory and chemokine pathways were significantly enriched in both GWAS studies, with contributing genes prioritized by PoPS including *IL2RA*, *IL23A*, *IL1R2*, *IL2*, *IL7R*, *IL6*, *MEF2A*, *PDGFRA*, *HLA* markers, and others (Tables S16 and S17). Interleukins are known modulators of angiogenesis and antiangiogenesis in tumors,^95^ and inhibition of interleukins has been found to suppress VEGF expression in tumors. Previous work on retinal vascular tortuosity in the UK Biobank also identified genetic loci linked to cardiometabolic diseases and cancer.^24^ Genetic contributors to microvascular indices may provide insights for therapeutic targets with pleiotropic effects for retinopathy, cancer, and microvascular disease in other tissues.

Fourth, mendelian randomization analysis allowed assessment of directionality of the links observed in the epidemiological analysis. Mendelian randomization uses human genetics for causal inference by leveraging the random assortment of genetic variants during meiosis at conception, which diminishes susceptibility to confounding or reverse causality.^96^ Here, we identified that individuals with genetically elevated blood pressure have lower retinal vascular density and FD. Similarly, individuals with genetically elevated risk for type 2 diabetes also had lower retinal vascular density, although no significant association was detected with FD, suggesting that the relationship with vascular density may be through vessel diameter as opposed to branching complexity, as supported by previous work on retinal vascular caliber.^14,16^ In addition, individuals predisposed to genetically lower vascular density have higher risk of myopia and higher risk of retinal detachments (independently of myopia and spherical equivalent) and higher risk of skin cancer (independently of principal components of genetic ancestry, self-reported skin color, and self-reported sun exposure and sun sensitivity). In particular, this genetic link between vascular density and retinal detachment, in addition to the phenotypic association of retinal vascular density with incident retinal detachment, highlights the likely causal link between these 2 phenotypes, thereby potentially identifying a new causal risk factor for retinal detachment that may be used for monitoring and therapeutic modulation.

Although our study has several strengths, there are important limitations to consider. First, it is possible that contributors to image quality, including the turbidity of the optical media, cataracts, and fundus pigmentation, may influence and confound the phenotypic and genotypic associations. However, we performed analyses conditioning on retinal conditions such as cataracts, retinal detachments, and myopia, as well as skin color, with largely unchanged associations with systemic traits. Second, although the TOPCON images are largely homogeneous in magnification, a range in image magnification exists that is correlated with an individual’s spherical equivalent. Sensitivity analyses adjusted for spherical equivalence and myopia indicated consistent associations. Third, given the paucity of accessible data sets with fundus images and genomic data and designation of retinal images as protected health information by the Health Insurance Portability and Accountability Act, we were unable to systematically replicate our GWAS results in adequately powered data sets. Previous GWASs of retinal vascular caliber performed in smaller studies^90,97,98^ identified overlapping loci with our present results at the *MEF2C*, *OCA2*, and *GNB3* loci. Fourth, although hypertension and diabetes may be the causal pathway from microvascular dysfunction to cardiovascular disease, it is possible that hypertension and diabetes are true confounders in associations with cardiovascular disease. Fifth, the present analysis was done with the UK Biobank, which is composed predominantly of Europeans, and had only fundus images acquired from TOPCON OCT scanner with limited retinal views. Further analyses in diverse ethnic cohorts and with other imaging modalities are necessary.

Overall, these findings support retinal microvascular indices as biomarkers for risk prediction and disease monitoring of systemic and ocular conditions. Furthermore, genome-wide association provided an unbiased assessment of the genes and biological pathways linked to the microvasculature. More research is needed to evaluate added benefit beyond existing clinical risk predictors and protocols and feasibility for incorporation into a clinical screening workflow. More broadly, our results illustrate the potential for using deep learning on retinal imaging to understand the microvasculature, with wide applications across diseases.

## Article Information

### Acknowledgments

UK Biobank analyses were conducted with Application 7089. The authors thank all study participants and staff for contributing to the UK Biobank Cohort.

### Sources of Funding

Dr Natarajan is supported by a Hassenfeld Scholar Award from the Massachusetts General Hospital and grants from the National Heart, Lung, and Blood Institute (R01HL1427, R01HL148565, and R01HL148050). S.M. Zekavat is supported by the National Health Institute National Heart, Lung, and Blood Institute (1F30HL149180-01) and the National Health Institute Medical Scientist Training Program Training Grant (T32GM136651). Dr O’Regan is supported by the Medical Research Council (MC-A658-5QEB0), National Institute for Health Research Imperial College Biomedical Research Centre, and British Heart Foundation (RG/19/6/34387, RE/18/4/34215). Dr Zebardast is supported by the National Eye Institute 1K23EY032634. Dr Wiggs is supported in part by National Eye Institute (R01EY020928, R01EY022305, R01EY031820, R01EY032559). The opinions expressed by the authors are their own and this material should not be interpreted as representing the official viewpoint of the National Institutes of Health.

### Disclosures

Dr Natarajan reports grants from Amgen during the conduct of the study and grants from Boston Scientific, grants and personal fees from Apple, personal fees from Novartis and Blackstone Life Sciences, and other support from Vertex outside the submitted work. Dr Ellinor has received grant support from Bayer AG and has served on advisory boards or consulted for Bayer AG, Quest Diagnostics, MyoKardia, and Novartis outside of the present work. Dr Wiggs has received grant support from Aerpio and served as a consultant for Allergan, Avellino, Editas, Maze, and Regenxbio outside of the present work. The other authors report no conflicts.

### Supplemental Material

Supplemental Methods

Supplemental Results

Figures S1–S9

Excel Files S1–S25

Reference 99

## Supplementary Material


