# Supplementary material for: Deep Learning of the Retina Enables Phenome- and Genome-Wide Analyses of the Microvasculature
Source: Circulation. 2021 Nov 8;145(2):134–50. doi: 10.1161/CIRCULATIONAHA.121.057709 (PMC8746912; doi:10.1161/CIRCULATIONAHA.121.057709)
Supplement: Supplementary file 1 [file cir-145-134-s001.pdf]

**SUPPLEMENTAL MATERIAL**

# Deep learning of the retina enables phenome- and genome-wide analyses of the microvasculature

Seyedeh Maryam Zekavat[1-4*], Vineet Raghu[3-5*], Mark Trinder[3,6], Yixuan Ye[2], Satoshi Koyama[3], Michael C. Honigberg[3,4], Zhi Yu[3], Akhil Pampana[3], Sarah Urbut[3,4], Sara Haidermota[4], Declan P. O'Regan[7], Hongyu Zhao[2,8], Patrick T. Ellinor[3,4], Ayellet V. Segrè[9], Tobias Elze[9], Janey L. Wiggs[9], James Martone[1], Ron A. Adelman[1], Nazlee Zebardast[9], Lucian Del Priore[1], Jay C. Wang[1], Pradeep Natarajan[3,4]

1. Department of Ophthalmology and Visual Science, Yale School of Medicine, New Haven, CT, USA
2. Computational Biology & Bioinformatics Program, Yale University, New Haven, CT, USA
3. Program in Medical and Population Genetics and Cardiovascular Disease Initiative, Broad Institute of MIT and Harvard, Cambridge, MA, USA
4. Cardiovascular Research Center, Massachusetts General Hospital, Harvard Medical School, Boston, MA, USA
5. Cardiovascular Imaging Research Center, Massachusetts General Hospital, Harvard Medical School, Boston, MA, USA
6. Centre for Heart Lung Innovation, The University of British Columbia, Vancouver, British Columbia, Canada
7. MRC London Institute of Medical Sciences, Imperial College London, London, UK
8. School of Public Health, Yale University, New Haven, CT, USA
9. Department of Ophthalmology, Massachusetts Eye and Ear, Harvard Medical School, Boston, MA, USA

* These authors equally contributed to this work.

Please address correspondence to:

Pradeep Natarajan MD, MMSc
Massachusetts General Hospital
185 Cambridge Street, CPZN 3.184
Boston, MA 02114
Office: 617-726-1843
Email: pnatarajan@mgh.harvard.edu
Twitter: @pnatarajanmd

**Table of Contents:**

A. **Supplemental Methods**
B. **Supplemental Results**
C. **Supplemental Figures**


1. Supplemental Figure 1: Machine learning for vessel segmentation
2. Supplemental Figure 2: External validation of deep learning-based segmentation approach on the Automated Retinal Image Analysis Dataset (N = 143).
3. Supplemental Figure 3: Relationship of left and right sided retinal vascular parameters with handedness.
4. Supplemental Figure 4: Relationship of retinal vascular parameters with baseline parameters. FD = fractal dimension.
5. Supplemental Figure 5: Phenome-wide associations of retinal vascular indices with prevalent disease
6. Supplemental Figure 6: Phenome-wide associations with quantitative clinical traits and biomarkers.
7. Supplemental Figure 7: Association of retinal vascular traits with incident HTN, with and without SBP and DBP adjustment from time of retinal fundus acquisition.
8. Supplemental Figure 8: Association of retinal vascular traits with incident T2D, with and without HbA1c adjustment from time of retinal fundus acquisition
9. Supplemental Figure 9: Association of retinal vascular FD and density with quantitative ocular traits.
10. Supplemental Figure 10: Phenome-wide associations of retinal vascular indices with incident disease additionally adjusted for prevalent type 2 diabetes mellitus and hypertension.
11. Supplemental Figure 11: Phenome-wide associations of retinal vascular indices with incident disease additionally adjusted for spherical equivalence
12. Supplemental Figure 12: Correlation of associations between genome-wide significant fine-mapped vascular density and FD loci
13. Supplemental Figure 13: Genome-wide association of retinal vascular density and FD excluding individuals with prevalent type 2 diabetes mellitus.
14. Supplemental Figure 14: Correlation of 173 independent genome-wide significant variants associated with vascular tortuosity with vascular density and FD.
15. Supplemental Figure 15: Gene prioritization by locus using PoPS for a) FD and b) vascular density
16. Supplemental Figure 16: Genetic correlation analyses and fine-mapping for retinal vascular density and FD
17. Supplemental Figure 17: Rare variant association analysis of loss of function and missense variants grouped by gene for retinal vascular a) FD and b) density.
18. Supplemental Figure 18: PheWAS of polygenic risk scores for a) vascular density and b) FD
19. Supplemental Figure 19: One-sample Mendelian Randomization for FD, vascular density PRS on myopia

**D. Supplemental Table Titles (in separate excel sheet)**

1. Supplemental Table 1: Ocular traits of images removed by outlier detection algorithm (left) and included in the study (right)
2. Supplemental Table 2: Baseline summary statistics from enrollment across the 54,813 individuals with fundus photos analyzed (post-quality control)
3. Supplemental Table 3: Univariate associations of FD and density with phenotypic covariates
4. Supplemental Table 4: Multivariate associations of FD and density with phenotypic covariates
5. Supplemental Table 5: LogReg PheWAS with prevalent events diagnosed before fundus image acquisition
6. Supplemental Table 6: Incident PheWAS using events diagnosed after time of image acquisition
7. Supplemental Table 7: linear regression across 88 quantitative clinical phenotypes
8. Supplemental Table 8: Adjustment of incident phewas for prevalent type 2 diabetes and hypertension
9. Supplemental Table 9: Adjustment of incident phewas for mean spherical equivalent
10. Supplemental Table 10: Genome-wide association study for FD (variants with P<1e-4). The effect allele is the alternate allele (second allele listed in column 'B').
11. Supplemental Table 11: Genome-wide association study for retinal vascular density (variants with P<1e-4). The effect allele is the alternate allele (second allele listed in column 'B').
12. Supplemental Table 12: Comparison of finemapped genome-wide significant loci across the retinal vascular density and FD associations
13. Supplemental Table 13: Prioritized genes from POPS
14. Supplemental Table 14: Genetic correlation analysis between GWAS x and GWAS y
15. Supplemental Table 15: Finemapped variants with rsIDs present for FD and retinal vascular density
16. Supplemental Table 16: Association of finemapped loci with other GWAS studies
17. Supplemental Table 17: FD FUMA Erichment analysis
18. Supplemental Table 18: Vascular Density FUMA Enrichment analysis
19. Supplemental Table 19: Rare variant burden analysis for retinal FD using rare (minor allele frequency<1%), missense deleterious or high-confidence loss-of-function variants
20. Supplemental Table 20: Rare variant burden analysis for retinal vascular density using rare (minor allele frequency<1%), missense deleterious or high-confidence loss-of-function variants
21. Supplemental Table 21: PheWAS of the FD and vascular density polygenic risk scores
22. Supplemental Table 22: 1-sample Mendelian randomization pheWAS of Vascular Density PRS PRS and FD PRS, after adjustment for self-reported skin color, sunlight exposure, and sunlight sensitivity
23. Supplemental Table 23: Genetic instrument for systolic blood pressure
24. Supplemental Table 24: Genetic instrument for diastolic blood pressure
25. Supplemental Table 25: Genetic instrument for type 2 diabetes mellitus

## Supplemental Methods:

### UK Biobank array genotyping and quality control

Genome-wide genotyping of blood-derived DNA was performed by UK Biobank across 488,377 individuals using two genotyping arrays sharing 95% of marker content: Applied Biosystems UK BiLEVE Axiom Array (807,411 markers in 49,950 participants) and Applied Biosystems UK Biobank Axiom Array (825,927 markers in 438,427 participants) both by Affymetrix (Santa Clara, CA)[43]. Variants used in the present analysis include those also imputed using the Haplotype Reference Consortium reference panel of up to 39 million bi-allelic variants and 88 million variants from the UK10K+1000 Genomes reference panels)[43]. Poor quality variants and genotypes were filtered as previously described[43], with additional filters including high-quality imputed variants (INFO score >0.4), minor allele frequency >0.005, and with Hardy-Weinberg Equilibrium $P \geq 1x10^{-10}$, as previously implemented using Hail-0.2 (https://hail.is/docs/0.2/index.html)[46-48]. Across all genetic analyses, we used data for participants with white British ancestry consenting to genetic analyses, with genotypic-phenotypic sex concordance, without sex aneuploidy, and after excluding one from each pair of 1$^{st}$ or 2$^{nd}$ degree relatives selected randomly.

### UK Biobank whole exome sequencing and quality control filters

UK Biobank whole exome sequencing was performed among 200,627 individuals using whole blood-derived DNA at the Regeneron Sequencing Center[44, 45], for which the methods have been previously described for the earlier release of data across approximately 50,000 individuals[44]. In brief, the IDT xGen Exome Research Panel v1.0 was used to capture exomes at over 20x coverage across 95% of sites. Extensive additional genotype, variant, and sample-level exclusion filters were applied to study high-quality autosomal exome sequence variants as previously done[45] using Hail-0.2.

In addition to any quality-control that was performed centrally, we applied extensive additional genotype, variant and sample quality-control procedures to ensure a high-quality dataset for analyses for both the common variant analyses using array genotyping data and rare variant analyses using whole exome sequencing data.

For the whole exome sequencing data used in rare variant analyses, we utilized the OQFE WES pVCF files provided by the UK Biobank, which contained calls for 200,643 sequenced samples. We applied genotype refinement to the raw genotype calls in the pVCF files using Hail-0.2 (https://hail.is/docs/0.2/index.html). We first split multi-allelic sites to represent separate bi-allelic sites.

We first performed genotype quality control, filtering out calls that did not pass the following hard filters were then set to no-call in our analysis:

- For homozygous reference calls: Genotype Quality < 20; Genotype Depth < 10; Genotype Depth > 200
- For heterozygous calls: (A1 Depth + A2 Depth)/Total Depth < 0.9; A2 Depth/Total Depth < 0.2; Genotype likelihood[ref/ref] < 20; Genotype Depth < 10; Genotype Depth > 200

- For homozygous alternative calls: (A1 Depth + A2 Depth)/Total Depth < 0.9; A2 Depth/Total Depth < 0.9; Genotype likelihood[ref/ref] < 20; Genotype Depth < 10; Genotype Depth > 200

These filters removed 9% of the 3,573,574,459,423 raw genotype calls leaving 3,214,727,581,104 genotype calls across 17,981,897 variant sites and 200,643 samples.

We then performed variant-level quality control. We removed variants that failed the following filters:

- Call rate of < 90% (restricting to males for Y chromosomal markers) (N= 4,023,284)
- Failed a liberal Hardy-Weinberg Equilibrium test (HWE) at $P < 10^{-14}$ among unrelated samples (not applied to Y chromosomal markers) (N=136,869)
- Present in Ensembl low-complexity regions (N=748,116)
- Monomorphic in the final dataset (N=55,614)

After performing these variant filters, 13,003,057 variants remained of which 12,756,075 were autosomal.

To perform sample level quality control, we computed a number of quality metrics to identify bad-quality or duplicated samples. We first used KING6 (version 2.2.5) to calculate pairwise heterozygote concordance rates for each pair of samples, using the high-quality independent autosomal markers. Then we used the high-quality autosomal variants present in both WES and array datasets to compute per-sample heterozygote concordance rates between WES calls and genotyping array calls. We inferred the genetic sex of each participants with the --check-sex option in PLINK, using the high-quality independent X-chromosomal markers. We set any sample with F > 0.8 to male, while samples with F < 0.5 were set to female. Finally, using all ~12.7M autosomal WES variants, we computed a number of additional metrics including sample call rate, transition/transversion ratio (Ti/Tv), heterozygote/homozygote ratio (Het/Hom), SNV/indel ratio (SNV/indel) and the number of singletons. After computing these metrics, we excluded participants based on the following criteria:

- Decided to revoke their consent
- Sample duplicates based on heterozygote concordance rates > 0.8 (N=0)
- Samples with blatant discordance between self-reported and genetically inferred sex
- Discordance between WES and array calls with heterozygote concordance rates < 0.8
- Call rate < 90%
- Samples further than 8 standard deviations from the mean for Ti/Tv (n=0), Het/Hom (N=100), SNV/indel (N=1) and number of singletons (N=111)

After applying these filters 200,337 samples remained for analysis.

**Automatic image outlier detection and removal**

To automatically identify poor quality fundus photographs, we developed a convolutional neural network model on the Google Cloud's AI platform. We used a sample of 1,000 retinal fundus photographs from the UK Biobank. We first included all images from those with multiple photos of the same eye from a single visit (to enrich for poor

quality images; 827 photos from 206 individuals). The remaining individuals were randomly sampled from all participants with fundus photography (additional 173 photos from 87 individuals). A board-certified ophthalmologist (J.C.W) hand-labeled images as poor quality or good quality based on presence or absence of motion artifact, media opacity, and ability to clearly distinguish retinal vessels on the photograph. The model was trained and tuned on a random sample of 80% of individuals (N=233 individuals, 794 photographs) and tested on the remaining 20% of individuals (N=60 individuals, 206 photographs). A 45% poor-quality probability was used as the threshold to remove poor quality images to balance removal of poor-quality images and sufficient sample size for downstream association studies, which resulted in removal rates similar to other published studies of UK Biobank Fundus photos[49, 50]. This threshold resulted in a sensitivity of 97.4% and a specificity of 100.0% for detecting poor quality images. The deep learning model was then applied to UK Biobank fundus photographs. Prior to analysis, a pixel intensity threshold was used to detect the outer edge of the fundus, and images were square cropped around the fundus boundary. For model development, images were resized to 320x320 pixels. Originally, 134,653 fundus photographs were available from 67,339 individuals from the UK Biobank enrollment visit. After applying the outlier detection algorithm and removing all photographs with >45% probability of poor quality, 99,736 images remained from 55,603 participants, resulting in removal of 26% of the original images.

**Overview of prior methods for retinal vessel segmentation:**

There has been an extensive body of work using automated computational approaches to segment retinal microvasculature and quantify microvasculature phenotypes[42, 51, 52] . These approaches can be broadly classified into unsupervised methods and supervised methods. Unsupervised approaches[53, 54] do not rely upon hand-drawn segmentations to train a classification model and instead use mathematical transformations of the raw image to identify edges corresponding to vessel walls, and then perform post-processing steps such as in-painting to fill in holes in segmented vasculature. Classical supervised approaches[55, 56] use transformations (e.g., wavelet filters)[55] to convert the raw pixel intensities into usable features which are then passed to a machine learning algorithm such as a Support Vector Machine or a Gaussian Mixture Model to classify each pixel as vasculature or not. More recently, studies have shown that deep neural networks substantially improve segmentation performance over classical approaches[52]. These models have an added benefit that once trained, they do not require any specific parameter settings during inference. However, it is not yet known whether deep learning-based segmentation approaches can generate quantifiable vascular phenotypes (e.g., fractal branching)[42] and whether these phenotypes are useful measures of disease risk.

**Deep learning model training process**

Our deep learning model consisted of an ensemble of 10 U-Net models each randomly initialized and trained for 1,000 epochs using this procedure. One-cycle learning rates were used with a maximum rate of 1e-4 during training. Geometric data augmentation was used during training including 5 degrees of random rotation, 50% brightness/contrast adjustment, 20% zoom in/out, random black-white inversion, and patch sampling to 25% of

original image size. The final segmentation mask was determined by rounding the pixel-wise probability of each model to 0/1 and taking a majority vote for each pixel.

**Fractal dimension and vascular density calculation**

The segmentation model was applied to these images to calculate fractal dimension and total vessel area. Fractal dimension was calculated using a box counting method[61] as previously described for the retina[42], and applied to the segmented vasculature. Vessel density was defined as the total number of pixels in the segmented vessels given a fixed dimension of 320x320 pixels for each image. For individuals with multiple photos from the same visit, the maximum fractal dimension and vessel density for each eye was used. Extreme outliers for right and left eye vascular FD and density were excluded by adjusting the traditional box and whisker upper and lower bounds and accounting for skewness in the phenotypic data using the Robustbase package in R (setting range=3) (https://cran.r-project.org/web/packages/robustbase/robustbase.pdf). For all analyses except for the quantitative ocular traits (where eye-specific analyses were performed), FD and vascular density were averaged across right and left eyes and inverse rank normalized to mean 0 and standard deviation 1. For the quantitative ocular traits, analyses were performed by eye and retinal vascular FD and density were inverse rank normalized to mean 0 and standard deviation 1 for right and left eyes separately.

**Phenome-wide association analyses (PheWAS)**

PheWAS with prevalent and incident phenotypes was performed across all of the 1,866 hierarchical phenotypes defined from the Phecode Map 1.2[62] ICD-9 (https://phewascatalog.org/phecodes) and ICD-10 (https://phewascatalog.org/phecodes_icd10) phenotype groupings[63]. Associations of retinal vascular FD and vascular density with prevalent phenotypes were performed utilizing logistic regression models, and associations with incident phenotypes were performed using Cox proportional hazards models after excluding individuals with the corresponding diagnosis at or prior to enrollment. Both models were adjusted for age, $age^2$, sex, smoking status (current/prior/never smoker), and ethnicity (Data field 21000). The proportional hazards assumption was assessed by Schoenfeld residuals and was satisfied for each model. Analysis was performed across disease phenotypes with at least 9 cases with retinal fundus images available. Statistical significance was defined using false discovery rate <0.05.

PheWAS across 88 quantitative systemic biomarkers acquired at enrollment including blood counts (Category ID 100081), blood biochemistry markers (Category ID 17518), liver MRI iron and inflammation measures (Category 126), arterial stiffness and reflection index from finger photoplethysmography (Category 100007), blood pressure (Category 100011), pulmonary function tests from spirometry (Category 100020), left ventricular size and function as well as pulse wave analysis from cardiac MRI (Category 102), as well as eye measures (Category 100011) were performed. For all phenotypes, sex-specific extreme outliers were excluded by adjusting the traditional box and whisker upper and lower bounds and accounting for skewness in the phenotypic data using the Robustbase package in R (setting range=3) as previously performed[46-48] (https://cran.r-

project.org/web/packages/robustbase/robustbase.pdf). Quantitative traits were inverse rank normalized to mean 0 and standard deviation 1. Analyses were done using linear regression in models adjusted for age, age$^2$, sex, smoking status (current/prior/never smoker), and ethnicity. Statistical significance was defined using false discovery rate <0.05.

**Genomic in silico analyses utilizing the GWAS summary statistics**

Using the GWAS summary statistics, SNP heritability analysis was performed using LD-score regression with LDSC-v1.0.1 (https://github.com/bulik/ldsc) and European LD scores from 1000 Genomes[65]. Fine-mapping to prioritize causal variants at each locus was performed using the FINEMAP-v1.4 software[66]. Putative causal genes were prioritized using PoPS software[67] (https://github.com/FinucaneLab/pops) which integrates GWAS summary statistics with gene expression, biological pathways, and predicted protein-protein interaction data to identify likely causal genes at each genome-wide significant locus. Enrichment analysis was performed using the FUMA and EnrichR web browsers across genes with PoPS z-score>1. Genetic correlation analysis was performed using GNOVA, using GWAS summary statistics from the datasets listed in **Supplemental Table 14** legend.

**Polygenic risk score (PRS) development and PRS-PheWAS**

Polygenic risk scores (PRS) were developed among individuals not included in the GWAS study (i.e., genotyped UK Biobank participants without retinal fundus images available). Significant, independent loci were identified using variants with $P<5x10^{-8}$, clumped in Plink-2.0 using an $r^2$ threshold of 0.1 across 1-MB genomic windows from the 1000 Genomes Project European reference panel. Additive PRSs for retinal vascular FD and, separately, vascular density were developed as such: $\sum_{i=1}^{N} Beta \times SNP_{ij}$ , where $Beta$ is the weight for each of the $N$ independent genome-wide significant variants in the GWAS, and $SNP_{ij}$ is the number of alleles (i.e., 0, 1, or 2) for $SNP_i$ carried by individual $j$ in the UK Biobank.

Further phenome-wide association of the retinal vascular FD and density PRS were performed to test the associations between genetically lowered retinal vascular FD and density associated with clinical traits in the UK Biobank, across combined prevalent and incident phenotypes from PheCode Map 1.2[62] ICD-9 and ICD-10 codes, adjusted for age, age$^2$, sex, ever smoking, the first ten principal components of genetic ancestry, and genotyping array. Given that several associations were identified with melanoma and skin cancers, additional sensitivity analyses were further performed adjusting for self-reported skin color (Field ID 1717), ease of skin tanning (Field ID 1727), time spent outdoors in summer and winter (Field ID 1050, 1060), and childhood sunburn occasions (Field ID 1737). Furthermore, given slight differences in magnification of the fundus images, which is correlated with degree of myopia (measured by spherical equivalent), we also performed sensitivity analyses adjusting for spherical equivalent, as done previously[64].

**1-sample Mendelian Randomization of blood pressure and type 2 diabetes on microvascular indices**

1-sample Mendelian randomization was performed using additive PRS for SBP (75 variants), DBP (75 variants), and type 2 diabetes mellitus (64 variants) comprised of genome-wide significant ($P<5x10^{-8}$), independent variants from European GWAS external to the UK Biobank, as done previously[47, 68] (**Supplemental Tables 23-25**). The PRS were inverse rank normalized then scaled such that each 1 unit increase in the SBP PRS reflected 10mmHg increase in SBP, and each 1 unit increase in DBP PRS reflected 10mmHg increase in DBP, and each 1unit increase in the type 2 diabetes PRS reflected a 2-fold increased risk of type 2 diabetes. These PRS were then associated with retinal vascular density and FD among unrelated individuals, in linear regression models adjusted for age, $age^2$, sex, smoking status, and principal components 1-10 of genetic ancestry.

**Rare variant association study (RVAS)**

RVAS was performed among unrelated individuals with both whole exome sequencing (WES) and retinal fundus images available using burden tests through the REGENIE package[69] (https://rgcgithub.github.io/regenie/options/). Exonic variants were filtered to rare (minor allele frequency <1%), predicted high-confidence loss-of-function by LOFTEE[71] or predicted missense deleterious by MetaSVM[70], and grouped by protein-coding gene. RVAS gene burden analysis was conducted, adjusting for age, $age^2$, sex, ever smoking, and the first ten principal components of genetic ancestry. Significance was defined based on a Bonferroni cutoff depending on the number of genes analyzed ($P<0.05/11,593$or $4.3x10^{-6}$ for vascular density; $P<0.05/12166$ or $4.1x10^{-6}$ for FD).

# Supplemental Results:

**Sensitivity analyses used in automated image quality control**

To ensure that the automated image removal model training process was robust to selection bias, we used 5-fold cross validation and found that the five models achieved an average AUC of 0.98 (±0.02), suggesting that selection bias was minor. To ensure that the quality control model was not detecting ocular pathology, we compared characteristics of poor-quality vs high quality images (**Supplemental Table 1).** Poor quality images were slightly enriched for glaucoma and cataracts – pathology that is expected to occlude vasculature, but were otherwise not meaningfully different.

**Baseline characteristics**

Across the 54,813 individuals analyzed, mean age was 56 (SD 8) years, 30,015 (55%) were female and 23,987 (44%) either previously or currently smoked (**Supplemental Table 2**). 50,223 (92%) self-reported ethnicity as White, 1,454 (2.7%) Black, 1,432 (2.6%) South Asian, 213 (0.4%) Chinese, and 1,491 (2.7%) as Other. Mean BMI was 27 (SD 4.7) kg/m$^2$, and 1,186 (2.2%) had prevalent type 2 diabetes mellitus, 14,968 (27.3%) hypertension, 2,693 (4.9%) coronary artery disease, 8,244 (15%) hypercholesterolemia, 188 (0.3%) chronic kidney disease, 731 (1.3%) stroke. Prevalence of clinical ocular conditions was low, with 2,015 (3.7%) having self-reported or diagnosed history of cataract, 802 (1.5%) glaucoma, 657 (1.2%) age-related macular degeneration, and 181 (0.3%) with diagnosed retinal detachment **(Supplemental Table 2**).

**Univariate and multivariate associations with retinal microvascular FD and density**

In univariate associations, average vascular FD and density across right and left eyes significantly decreased with age (**Figure 1c**), with slightly lower values present in females and individuals of Chinese or South Asian ethnicity, and slightly higher values present in current smokers (**Supplemental Figure 4, Supplemental Table 3**). Multivariate associations recapitulated the importance of including age, sex, current smoking status, and ethnicity in predicting retinal vascular FD and density (**Supplemental Table 4**). In multivariate models, female sex was associated with reduced FD but no difference was observed in vascular density compared to males. However, ancestral differences were more evident for vascular density than FD.

**Phenome-wide association study sensitivity analyses**

Sensitivity analyses were performed first adjusting the incident associations for prevalent hypertension and diabetes (**Supplemental Figure 10, Supplemental Table 8**), removing associations with most incident cardiovascular conditions (including incident heart failure and hypertensive heart disease); however a significant association remained for renal dialysis (Pre-adjustment: HR 1.61, P=1.99x10$^{-5}$; post-adjustment: HR 1.53, P=0.00019); however incident ocular phenotypes persisted after adjustment. Separately, analyses were performed adjusting the incident

associations for mean spherical equivalent (**Supplemental Figure 11, Supplemental Table 9**), identifying consistent association with incident ocular phenotypes after these adjustments.

**GWAS Sensitivity analyses and comparison with vascular tortuosity variants**

Further analyses removing prevalent type 2 diabetes mellitus cases identified consistent associations across all previously identified genome-wide significant loci (**Supplemental Figure 13, Supplemental Table 12**). Furthermore, a comparison was performed between 173 independent genome-wide significant variants[24] identified in a prior published GWAS of retinal vascular tortuosity and FD and density (**Supplemental Figure 14**). Significant, positive but not perfect genetic correlations were identified between vascular tortuosity and density ($R_{Pearson}$=0.44, P=7.7x$10^{-10}$) as well as FD ($R_{Pearson}$=0.24, P=0.0015), with several loci showing consistent effects between tortuosity and density (at *IRF4*:rs12203592, *FLT1*:rs17066617) and tortuosity and FD (at *IRF4*:rs12203592, *OCA2*:rs7164220, *HERC2*:rs12913832, *AGFG2*:rs187300659, *ZKSCAN1*:rs188746966), while several variants had heterogeneous effects, particularly at the *PDE3A* locus.

**Rare variant association study**

Rare variant association study (RVAS) was performed using 25,674 individuals with whole exome sequencing data, grouping together rare (minor allele frequency<1%), disruptive (i.e., MetaSVM[70] missense deleterious or high-confidence loss of function variants[71]) variants by gene (**Supplemental Figure 17**, **Supplemental Table 19,20**). While no exome-wide significant (i.e., P<4x$10^{-6}$) signal was attained, several suggestive associations were observed. Firstly, the top gene in the FD RVAS and one of the top genes of the retinal density RVAS analyses is *MITF* (melanocyte inducible transcription factor, also called microphthalmia associated transcription factor) (Beta -0.27, P=3.3x$10^{-5}$), whose signal was driven by a predicted deleterious missense variant with allele frequency 0.0032, rs149617956-A (p.Glu419Lys), which was suggestively associated with reduced vascular density (Beta -0.27 SD, P=9.4x$10^{-6}$) as well as FD (Beta -0.28 SD, P=2.5x$10^{-6}$) in the GWAS analyses. Second, for the vascular density RVAS, nominal association was detected with *GNB3* (Beta = -0.86SD, P=0.0064), which also had a more common missense variant with allele frequency 0.071 detected in the common variant GWAS as noted earlier, not included in this RVAS aggregation. Prior studies have linked *GNB3* to myopia[92], hypertension[91], as well as retinal vascular caliber[90], and retinal degeneration[99].

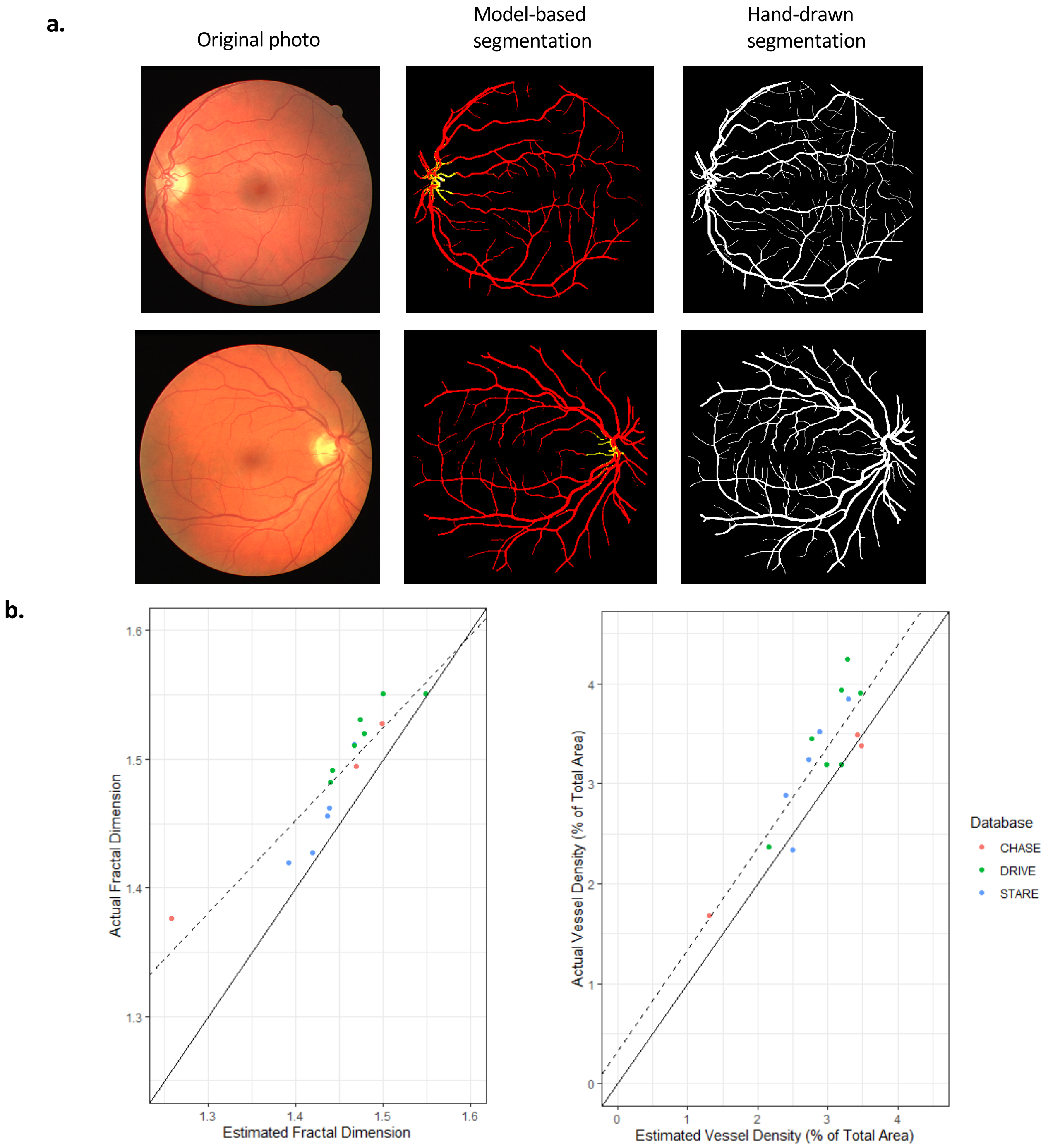


**Supplementary Figure 1: Machine learning for vessel segmentation. a.** showing two representative photos and the deep learning model-based segmentation versus hand-drawn segmentation. **b.** correlation between estimated and actual Fractal Dimension and vessel density from the testing data points across the CHASE, DRIVE, and STARE databases. DRIVE = Digital Retinal Images for Vessel Extraction (photographs from a diabetic retinopathy screening program in the Netherlands of subjects 25-90 years of age (https://drive.grand-challenge.org/); CHASE = Child Heart and Health Study in England (retinal fundus photographs of 9- and 10-year old children of different ethnic origin from England); STARE = the Structured Analysis of the Retina database (extracted from clinical visits to the Shiley Eye Center at the University of California, San Diego)

a.

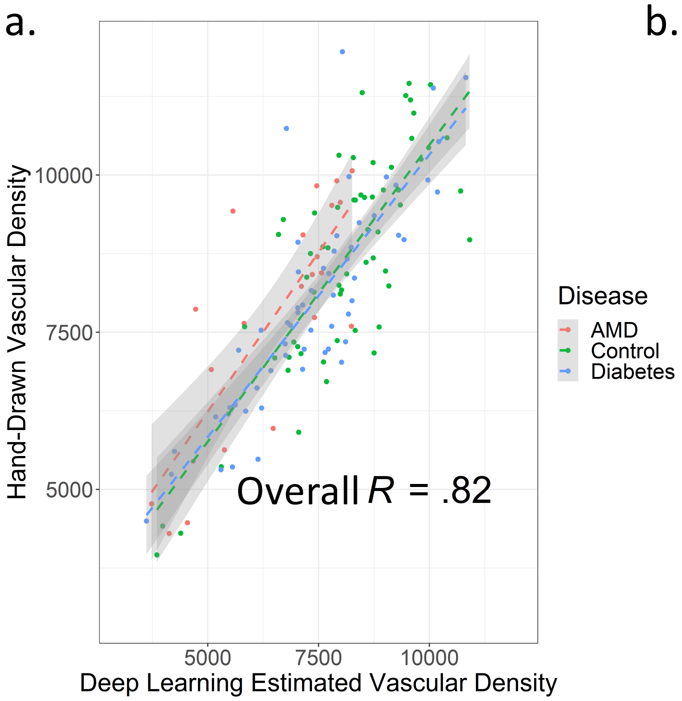


b.

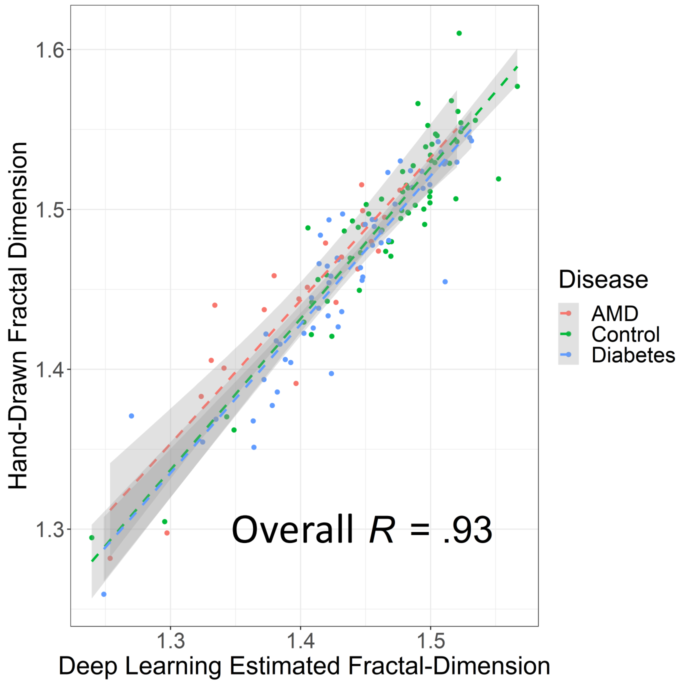


c.

| Approach | Accuracy | AUC | Reference |
|---|---|---|---|
| Deep learning (U-Net Ensemble) | **95.6%** | **97.4%** | Current manuscript |
| Azzopardi et al. 2015 | 94.0% | 89.2% | Azzopardi G, Strisciuglio N, Vento M and Petkov N. Trainable COSFIRE filters for vessel delineation with application to retinal images. *Med Image Anal*. 2015;19:46-57. |
| Zhao et al. 2015 | 94.0% | 84.0% | Zhao Y, Liu Y, Wu X, Harding SP, Zheng Y. Retinal vessel segmentation: An efficient graph cut approach with retinex and local phase. *PLoS One*. 2015;10:e0122332. |
| Bankhead et al. 2012 | 93.8% | 85.7% | Bankhead P, Scholfield CN, McGeown JG and Curtis TM. Fast retinal vessel detection and measurement using wavelets and edge location refinement. *PLoS One*. 2012;7:e32435. |
| Nguyen et al. 2013. | 93.8% | 87.5% | Nguyen UTV, Bhuiyan A, Park LA. An effective retinal blood vessel segmentation method using multi-scale line detection. *Pattern Recognition*. 2013;46:703-715 |
| Soares et al. 2006 | 93.6% | 90.7% | Soares JV, Leandro JJ, Cesar RM, Jelinek HF and Cree MJ. Retinal vessel segmentation using the 2-D Gabor wavelet and supervised classification. *IEEE Transactions on medical Imaging*. 2006;25:1214-1222. |
| Sofka et al. 2006 | 93.3% | 86.3% | Sofka M and Stewart CV. Retinal vessel centerline extraction using multiscale matched filters, confidence and edge measures. *IEEE transactions on medical imaging*. 2006;25:1531-1546. |

**Supplementary Figure 2**: **External validation of deep learning-based segmentation approach on the Automated Retinal Image Analysis Dataset (N = 143)**. a) Scatterplot of estimated and ground-truth vascular density by presence of disease in photo. Overall correlation is reported across all disease groups together. b) Scatterplot of estimated and ground-truth fractal dimension, and c) Comparison of deep learning-based segmentation with other image processing-based techniques.

**a.**

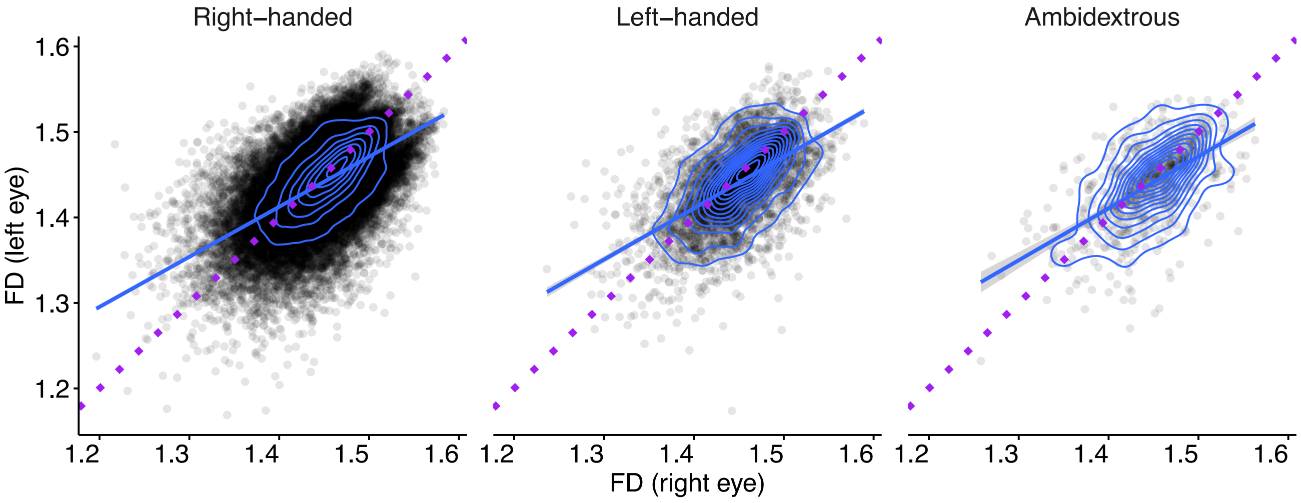


**b.**

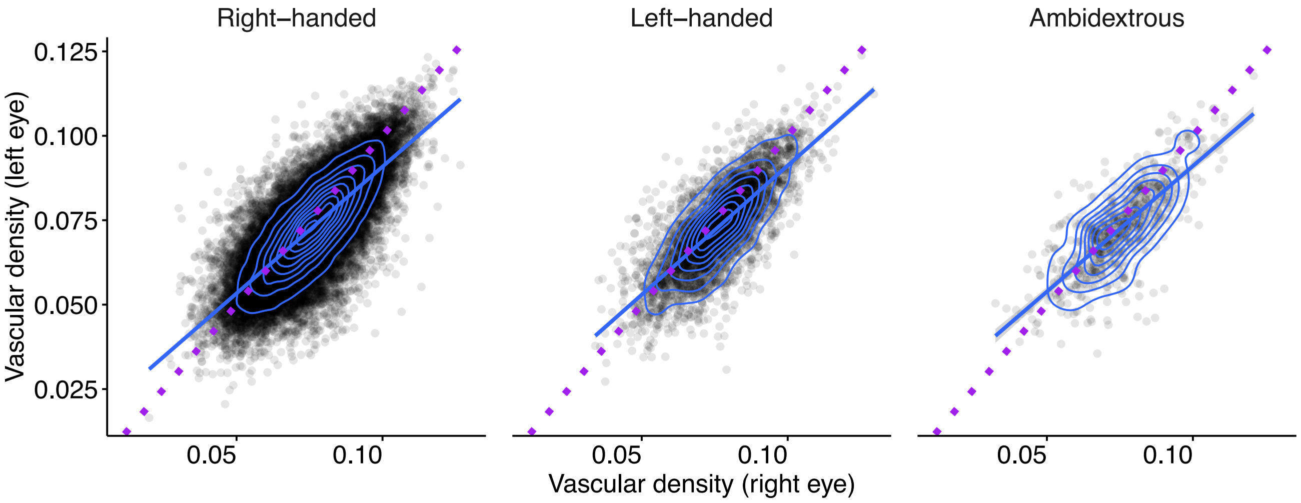


**Supplementary Figure 3: Relationship of left and right sided retinal vascular parameters with handedness**. The blue line and contours reflect the best fit of the data. The dotted purple line reflects the unity line (y=x). FD = fractal dimension.

**a.**

**b.**

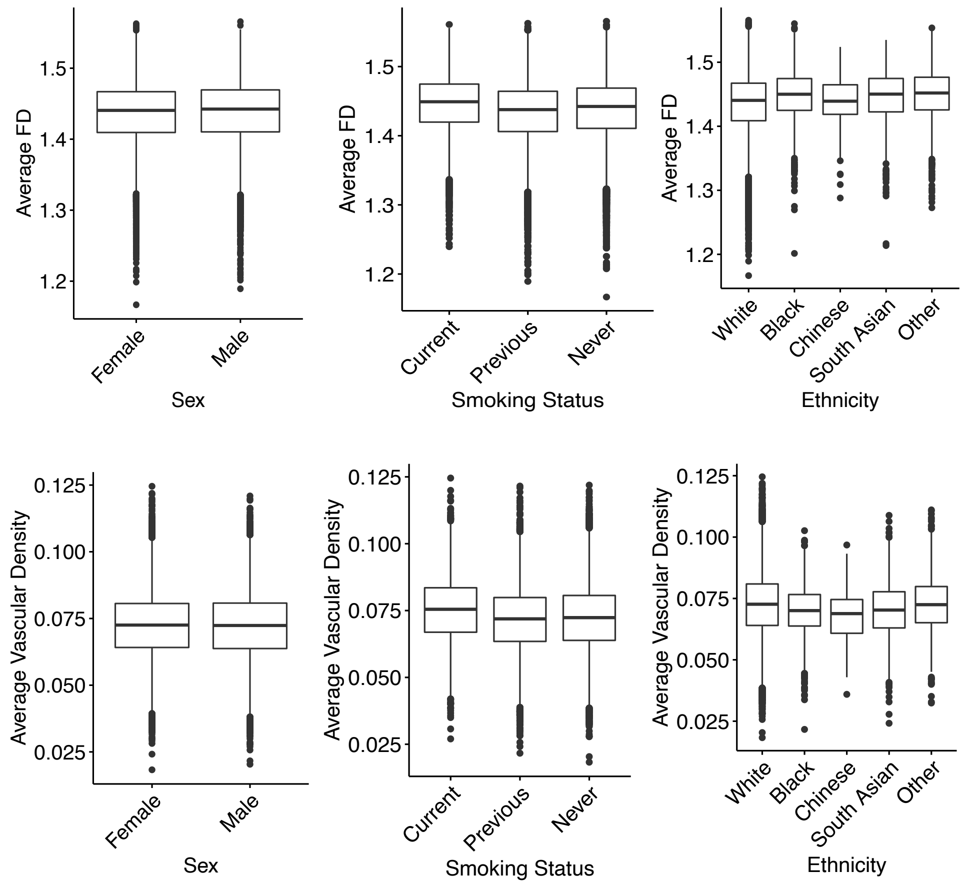


**Supplementary Figure 4: Relationship of retinal vascular parameters with baseline parameters. FD = fractal dimension.**

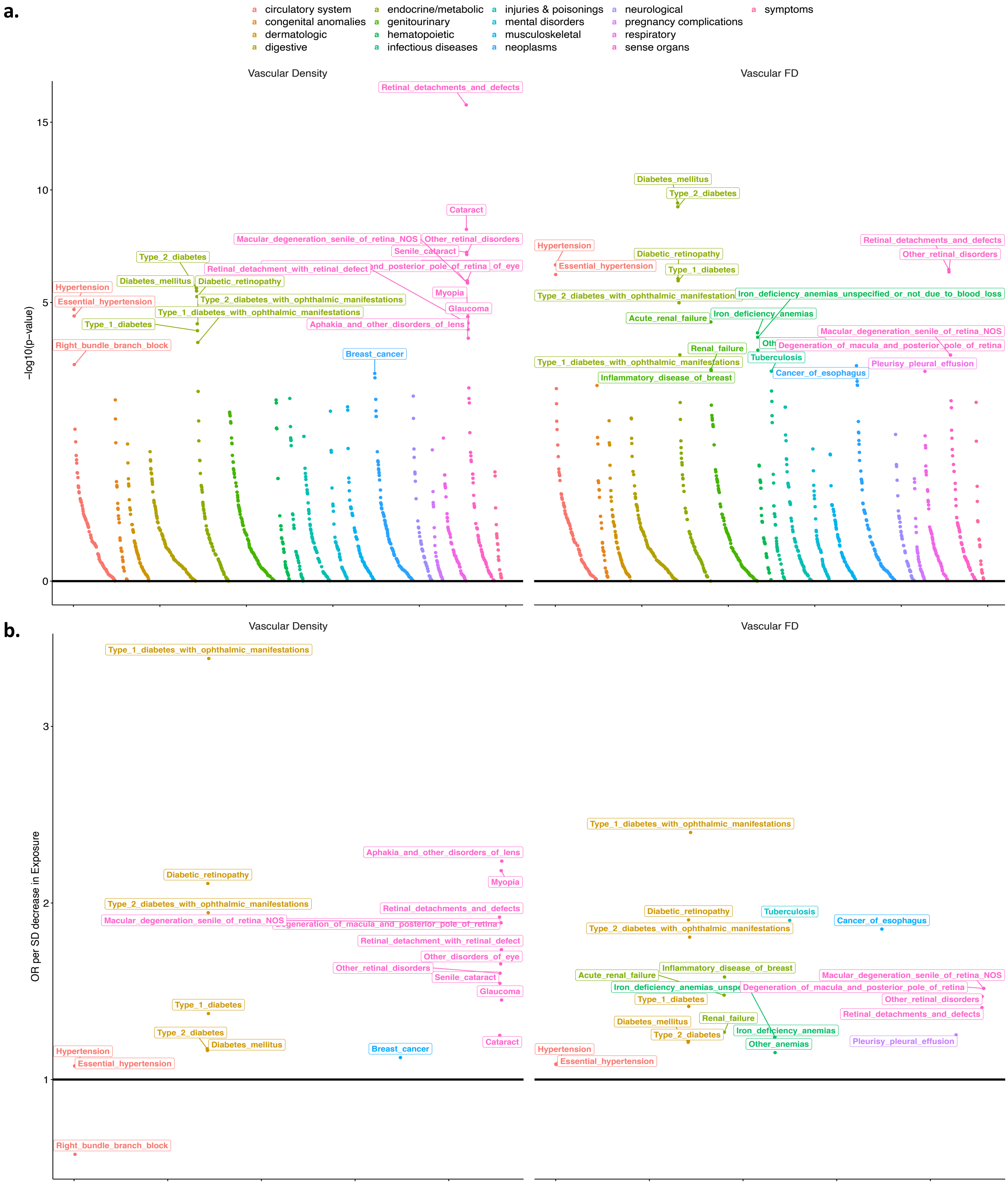


**Supplementary Figure 5: Phenome-wide associations of retinal vascular indices with prevalent disease.** Adjustment was performed for age, age², sex, smoking status, and ethnicity. **a.** –log10(P-value) of associations of retinal vascular density and FD with incident disease plotted grouped by phenotypic category. **b.** Odds ratio (OR) per 1 SD decrease in either vascular density (left) or FD (right). Labeled phenotypes have false discovery rate P<0.05. X-axis reflects an organized grouping of the phenotypes by phenotypic category and p-value of association. FD = fractional dimension.

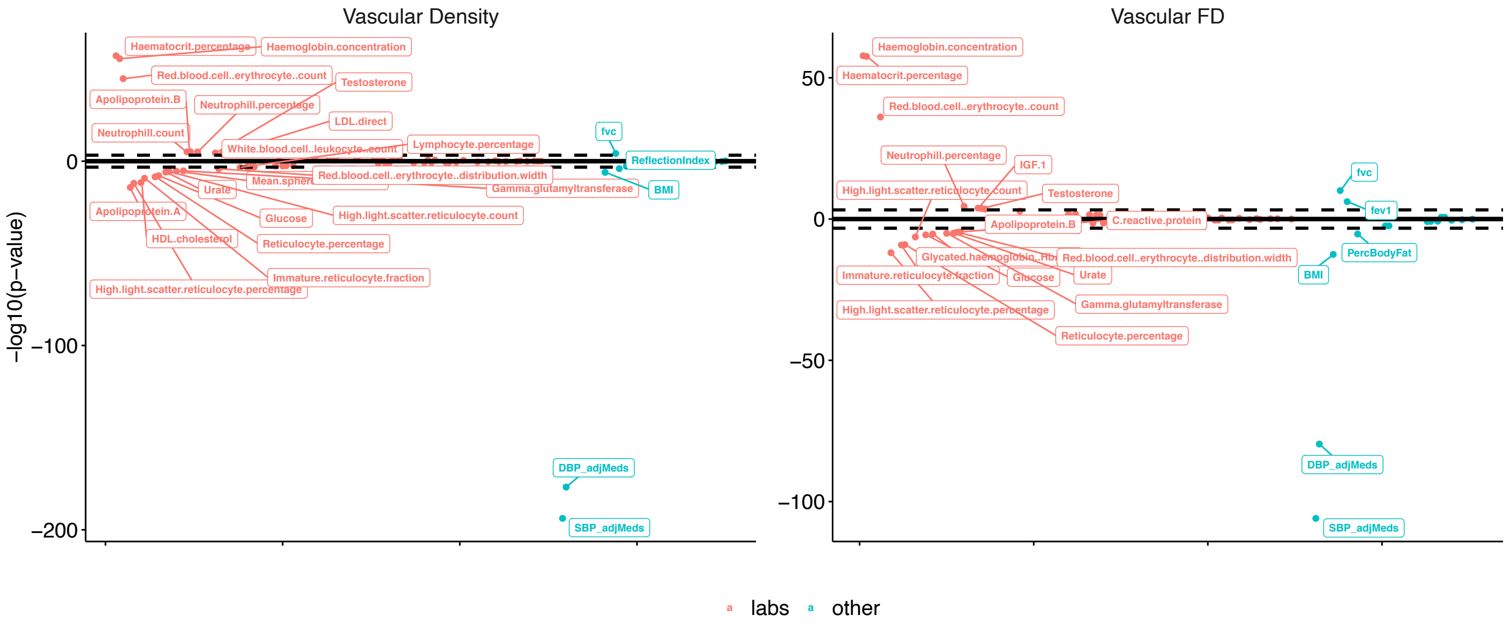


**Supplementary Figure 6: Phenome-wide associations with quantitative clinical traits and biomarkers.** Association of quantitative blood biomarkers and other quantitative clinical traits with vascular density (left) and FD (right). Linear regression models were used for analysis, adjusting for age, age$^2$, sex, smoking status, and ethnicity. Y-axis reflects the –log10(p-value) x beta direction, whereby above 0 reflects a positive beta (ie: each SD increase in phenotype is associated with increased vascular density or FD) and below 0 reflects a negative beta (ie: each SD increase in phenotype is associated with decreased vascular density or FD). Horizontal dotted line reflects the Bonferroni threshold for significance. X-axis reflects an organized grouping of the phenotypes by phenotypic category (laboratory values versus other quantitative phenotype) and p-value of association.

Fvc = forced vital capacity, BMI = body mass index, DBP_adjMeds = diastolic blood pressure, adjusted for antihypertensive medication use (see methods), SBP_adjMeds = systolic blood pressure, adjusted for antihypertensive medication use

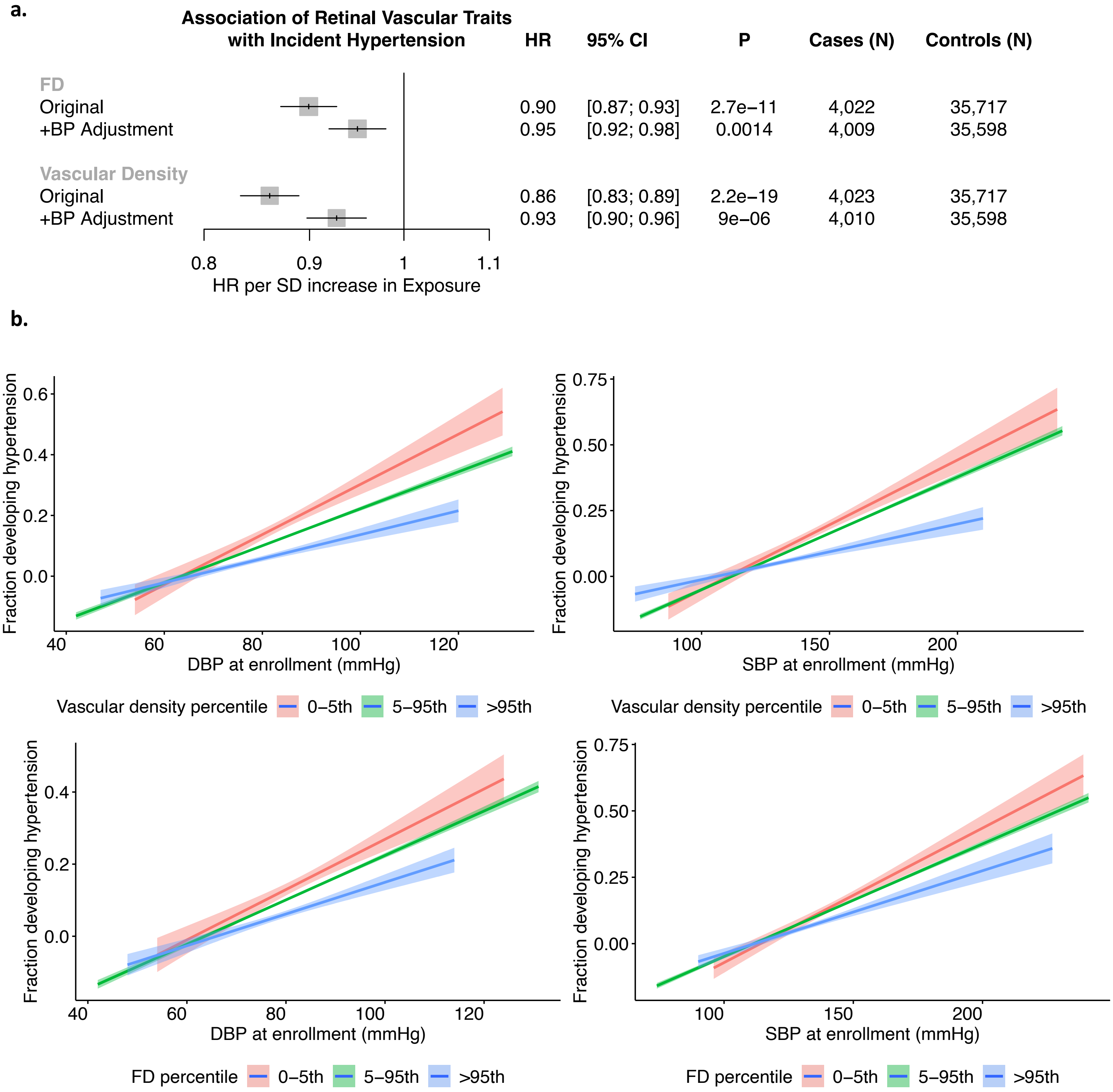


**Supplementary Figure 7: Association of retinal vascular traits with incident HTN, with and without SBP and DBP adjustment from time of retinal fundus acquisition. a)** Original Association of retinal vascular traits with incident T2D. "Original" model refers to adjustment for age, age$^2$, sex, smoking status, and ethnicity, and "+BP Adjustment" refers to additionally adjusting for SBP and DBP from time of retinal fundus acquisition. **b)** Incident hypertension risk conferred by DBP and SBP measured at enrollment, stratified by vascular density and FD. Shaded regions reflect the 95% confidence interval using a restricted maximum likelihood (REML) binomial generalized additive model with integrated smoothness (from the gam() function in R).
DBP = diastolic blood pressure, SBP = systolic blood pressure, FD = fractal dimension

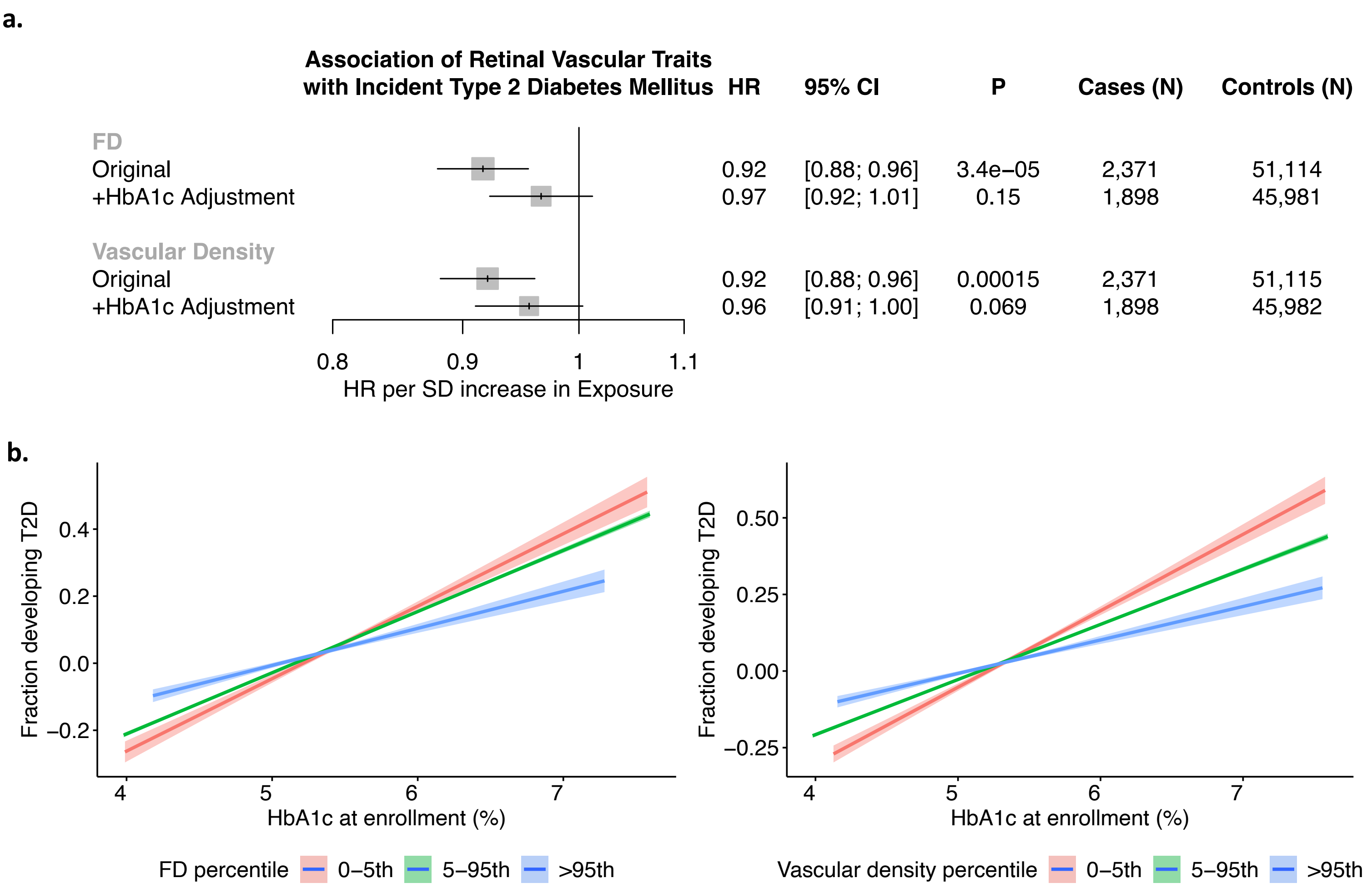


**Supplementary Figure 8: Association of retinal vascular traits with incident T2D, with and without HbA1c adjustment from time of retinal fundus acquisition**. a) Original Association of retinal vascular traits with incident T2D. "Original" model refers to adjustment for age, age$^2$, sex, smoking status, and ethnicity, and "+HbA1c Adjustment" refers to additionally adjusting for HbA1c from time of retinal fundus acquisition. b) Incident type 2 diabetes risk conferred by HbA1c measured at enrollment, stratified by vascular density and FD percentiles. Shaded regions reflects the 95% confidence interval using a restricted maximum likelihood (REML) binomial generalized additive model with integrated smoothness (from the gam() function in R).
FD = fractal dimension; T2D = type 2 diabetes mellitus

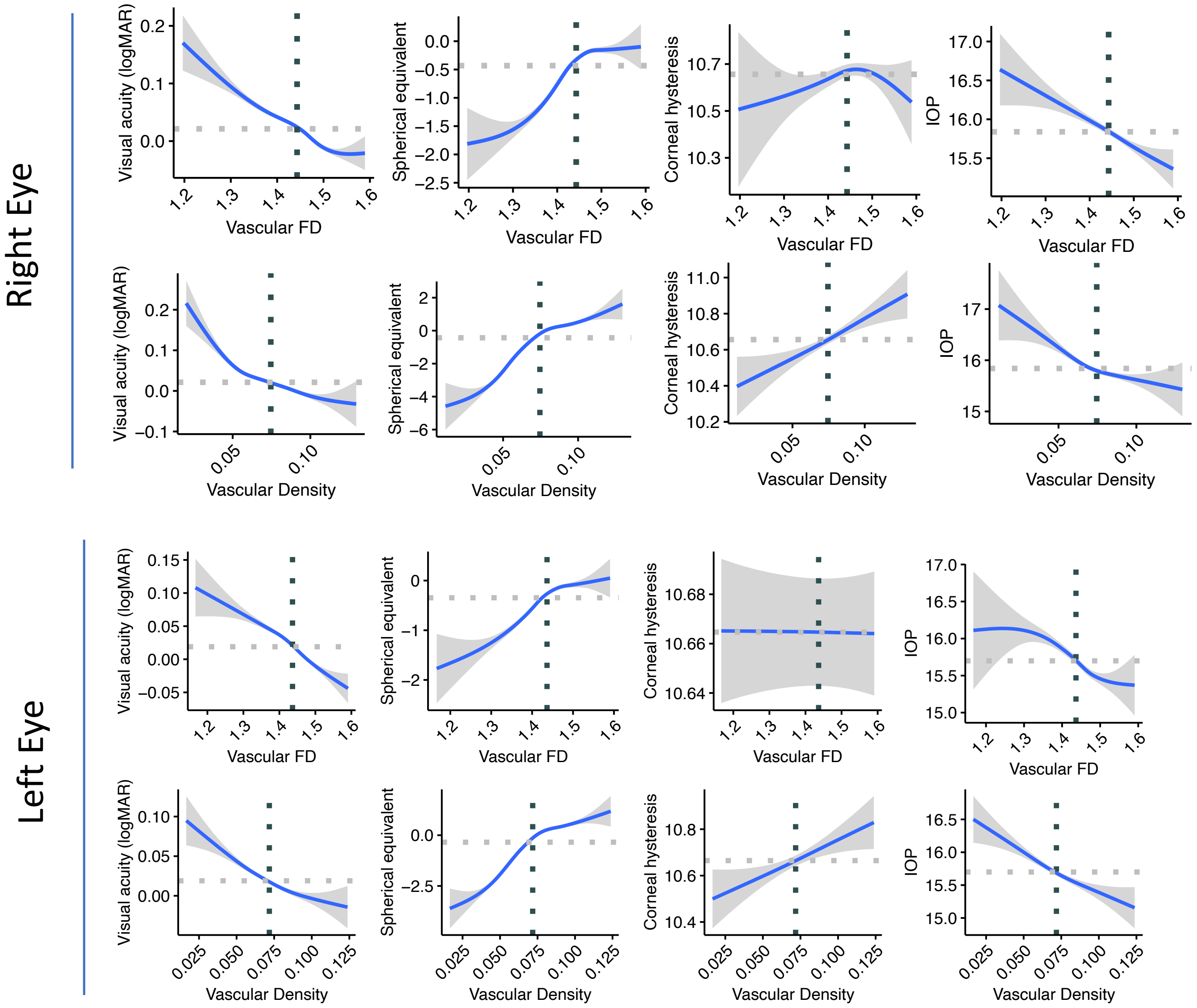


**Supplementary Figure 9: Association of retinal vascular FD and density with quantitative ocular traits.** Associations were performed separately by eye. Horizontal and vertical dotted lines represent the mean values of the corresponding trait and are provided for reference. Shaded grey region reflects the 95% confidence interval using a restricted maximum likelihood (REML) generalized additive model with integrated smoothness (from the gam() function in R). IOP = intraocular pressure; FD = fractal dimension; logMAR = logarithm of the minimal angle of resolution.

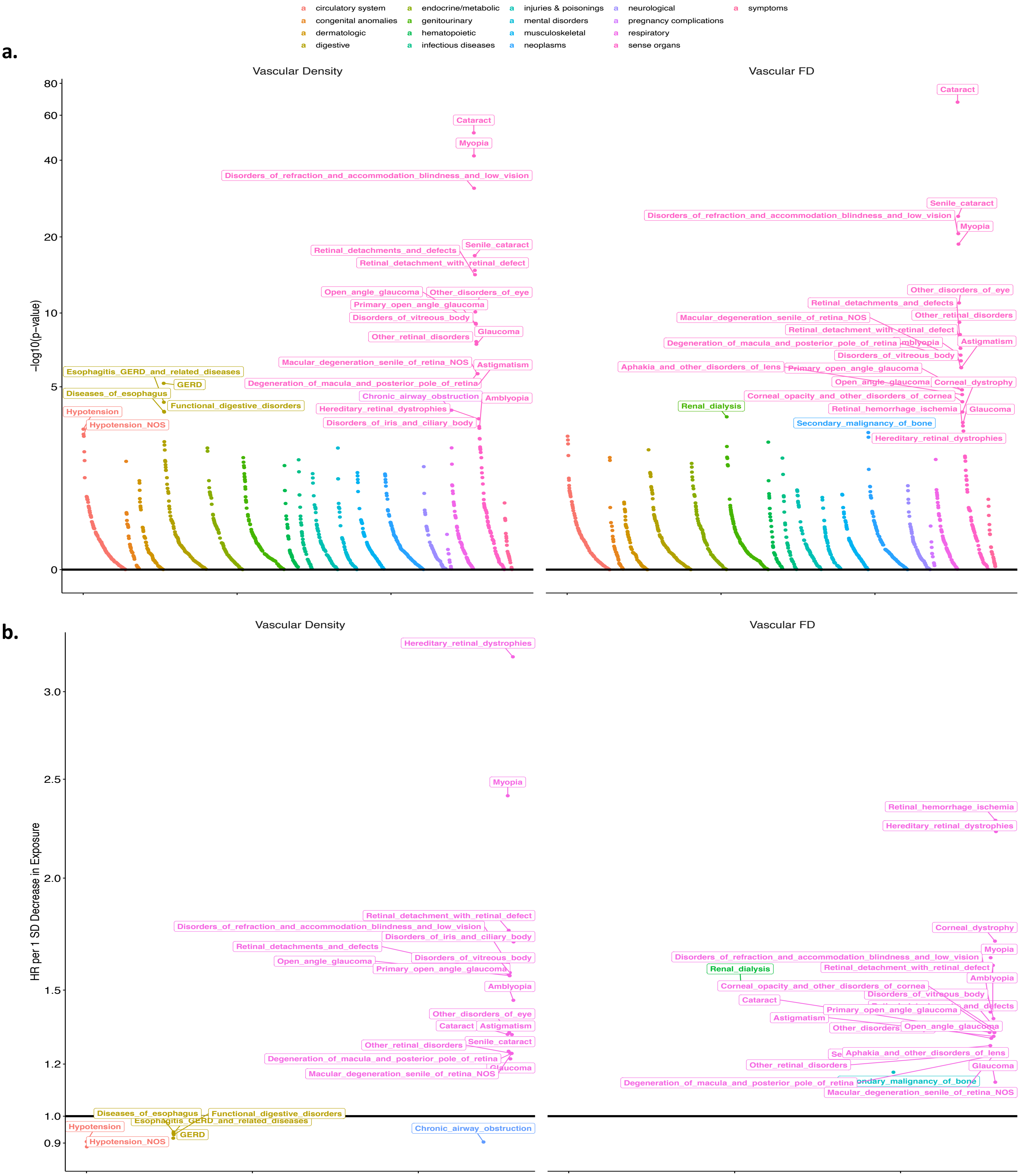


**Supplementary Figure 10: Phenome-wide associations of retinal vascular indices with incident disease additionally adjusted for prevalent type 2 diabetes mellitus and hypertension**. Adjustment was performed for age, age$^2$, sex, smoking status, ethnicity, type 2 diabetes mellitus, and hypertension. a) –log10(P-value) of associations of retinal vascular density and FD with incident disease plotted grouped by phenotypic category. b. Hazard ratio (HR) per 1 SD decrease in either vascular density (left) or FD (right). Labeled phenotypes have false discovery rate P<0.05. X-axis reflects an organized grouping of the phenotypes by phenotypic category and p-value of association. FD = fractal dimension, HR = hazard ratio

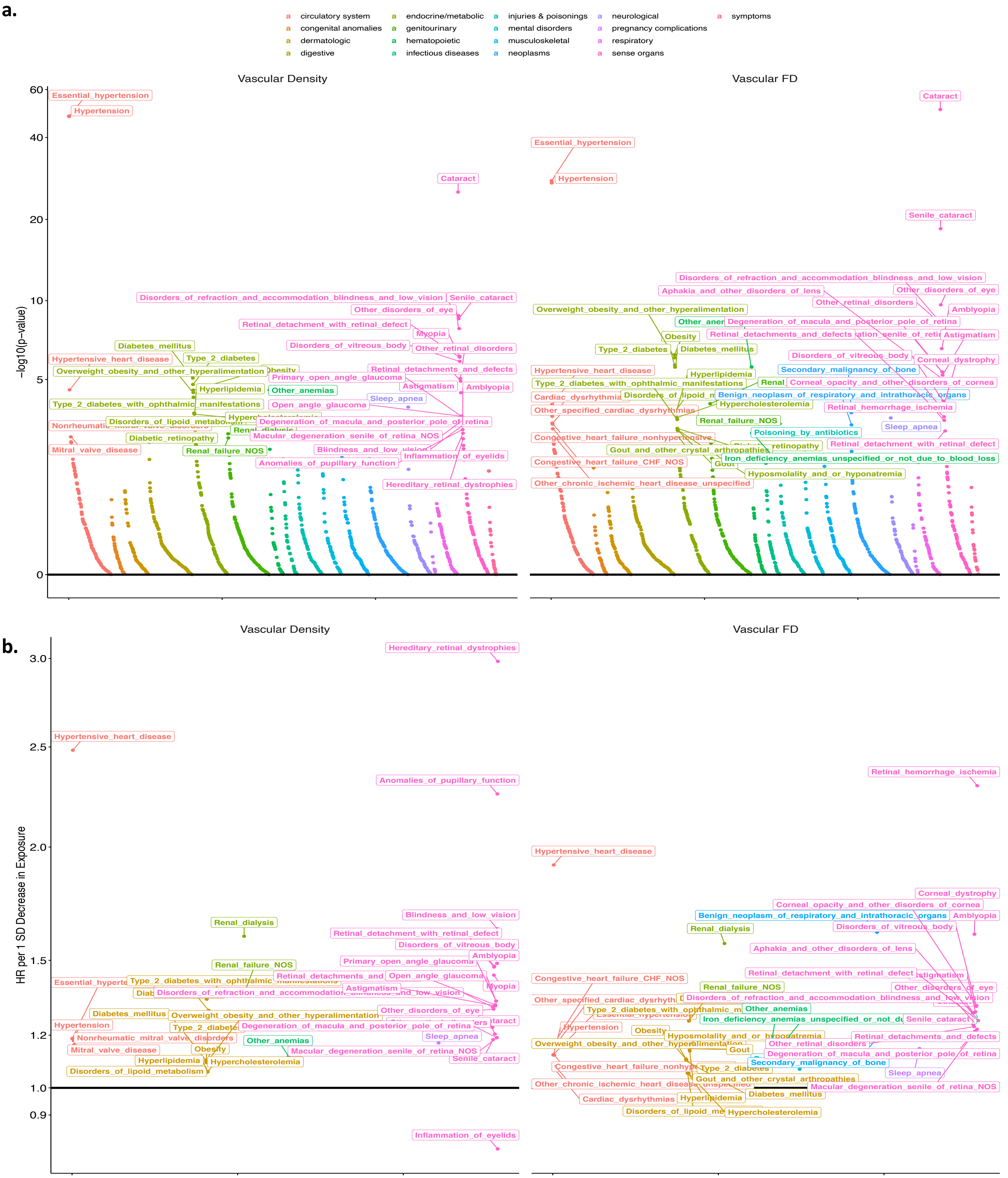


**Supplementary Figure 11: Phenome-wide associations of retinal vascular indices with incident disease additionally adjusted for spherical equivalence.** Adjustment was performed for age, age², sex, smoking status, ethnicity, and spherical equivalence averaged across both eyes. a) –log10(P-value) of associations of retinal vascular density and FD with incident disease plotted grouped by phenotypic category. b. Hazard ratio (HR) per 1 SD decrease in either vascular density (left) or FD (right). Labeled phenotypes have false discovery rate P<0.05. X-axis reflects an organized grouping of the phenotypes by phenotypic category and p-value of association.

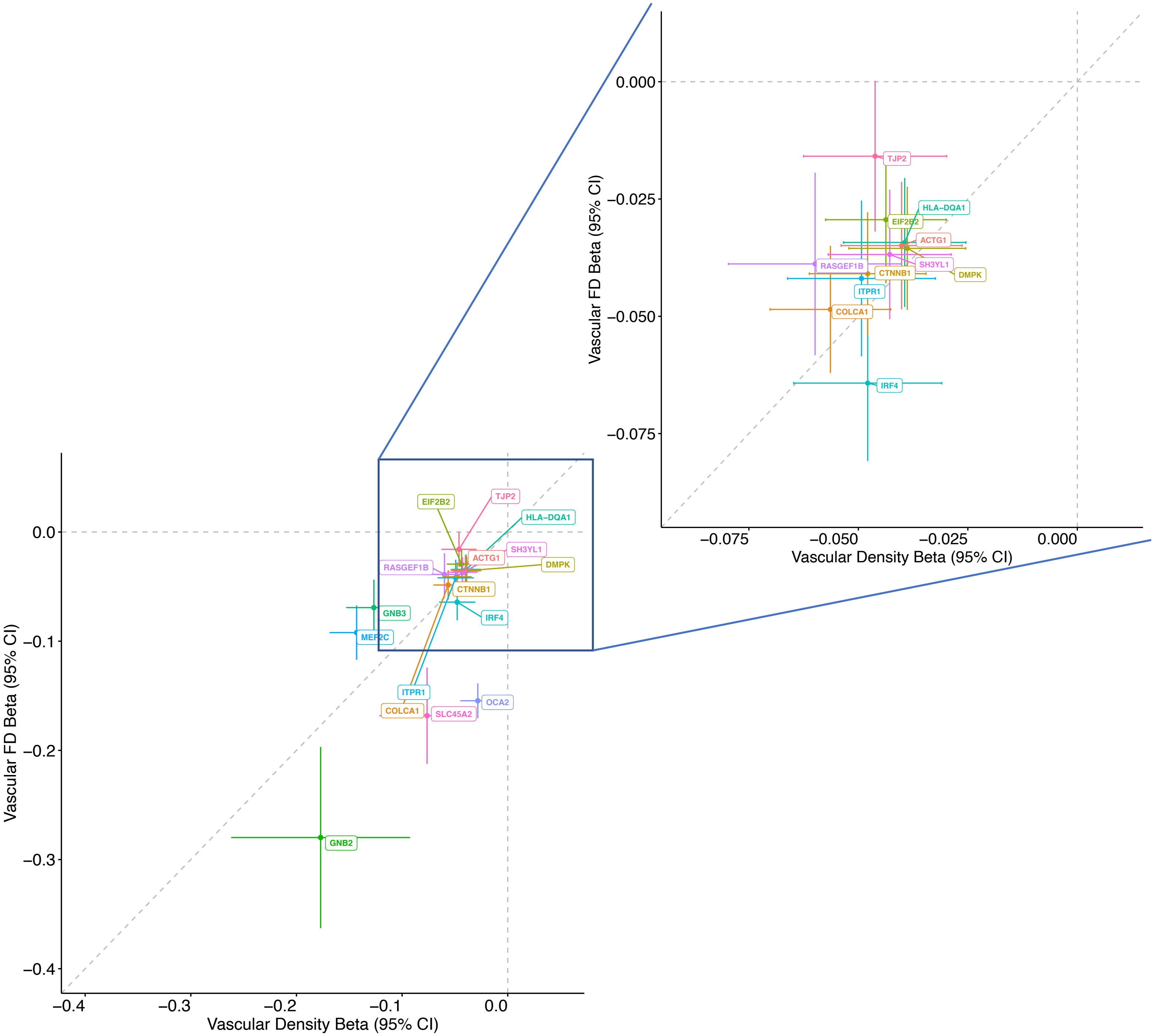


**Supplementary Figure 12: Correlation of associations between genome-wide significant fine-mapped vascular density and FD loci.** Variants are labeled by their prioritized PoPS gene at that locus (presented in Extended Data Figure 7). A list of the variants and the exact summary statistics presented in this figure is available in Supplementary Table 11. A zoomed-in version of plot **(a)** is provided in panel **(b).** FD = fractal dimension

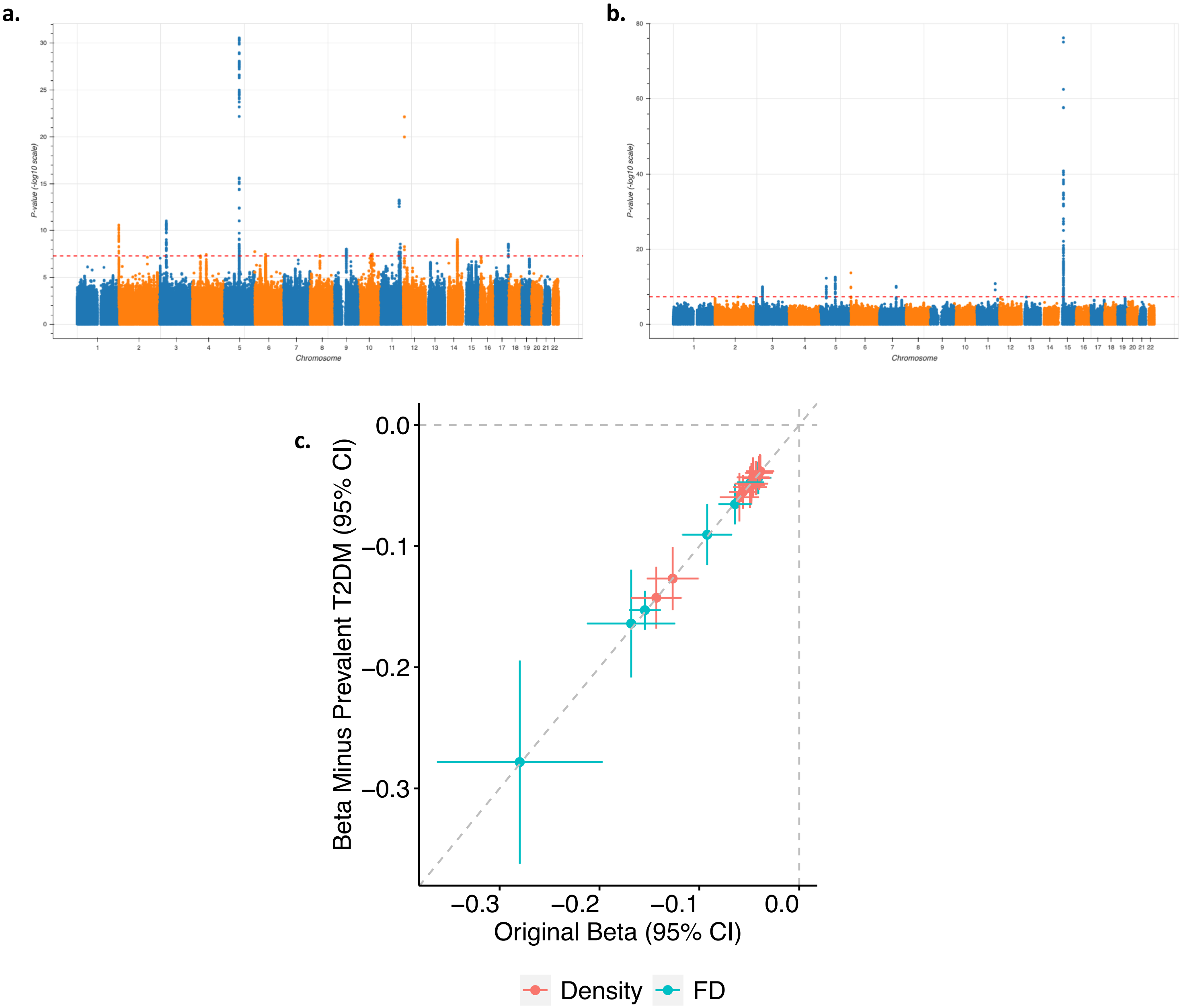


**Supplementary Figure 13: Genome-wide association of retinal vascular density and FD excluding individuals with prevalent type 2 diabetes mellitus.** Genome-wide association study of **a.** retinal vascular density and **b.** fractal dimension after removing individuals with prevalent type 2 diabetes mellitus (T2DM) from the analyses (final sample size, N=38,109). Comparison of betas and 95% confidence intervals for the fine-mapped genome-wide significant variants (listed in **Supplementary Table 12**) across the original GWAS compared to the GWAS after removing prevalent T2DM cases shows strong correlation of betas for all loci ($R_{Pearson}$=0.9997). Oblique dotted line reflects y=x. FD = fractal dimension

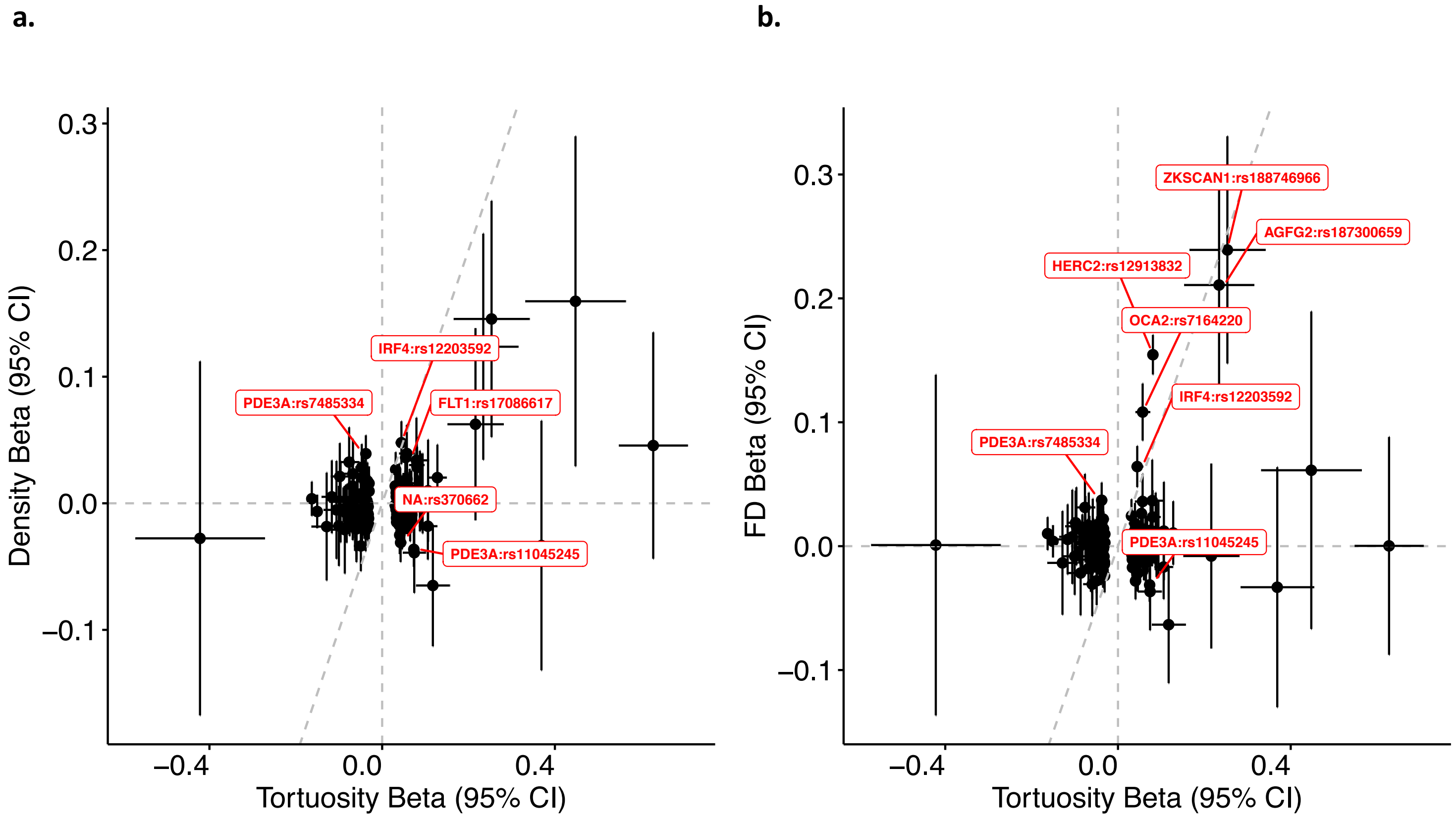


**Supplementary Figure 14: Correlation of 173 independent genome-wide significant variants associated with vascular tortuosity with vascular density and FD.** Correlation of betas from 173 independent variants associated with vascular tortuosity (from Tomasoni et al. MedRxiv 2021) with retinal vascular a) density ($R_{Pearson}$=0.44, P=7.7e-10) and b) FD ($R_{Pearson}$=0.24, P=0.0015). Oblique dotted line reflects y=x, horizontal and vertical dotted lines reflect beta=0. Variants with P<1e-4 for Density (a) and FD (b) are labeled, along with the closest gene. FD = fractal dimension

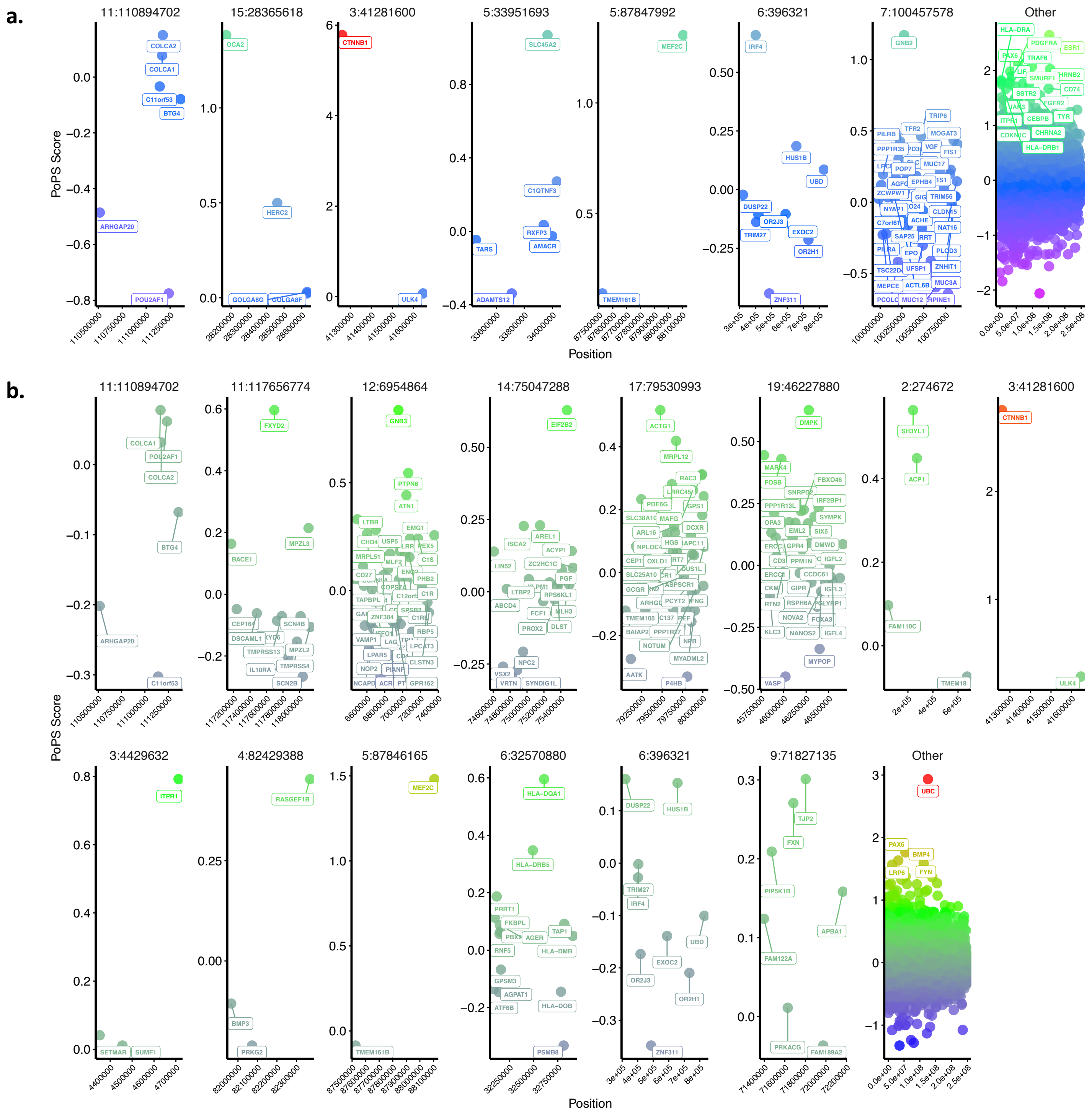


**Supplementary Figure 15: Gene prioritization by locus using PoPS for a) FD and b) vascular density.** X-axes reflect chromosomal location, y-axes and colors reflect PoPS z-scores. PoPS = Polygenic Priority Score[56].

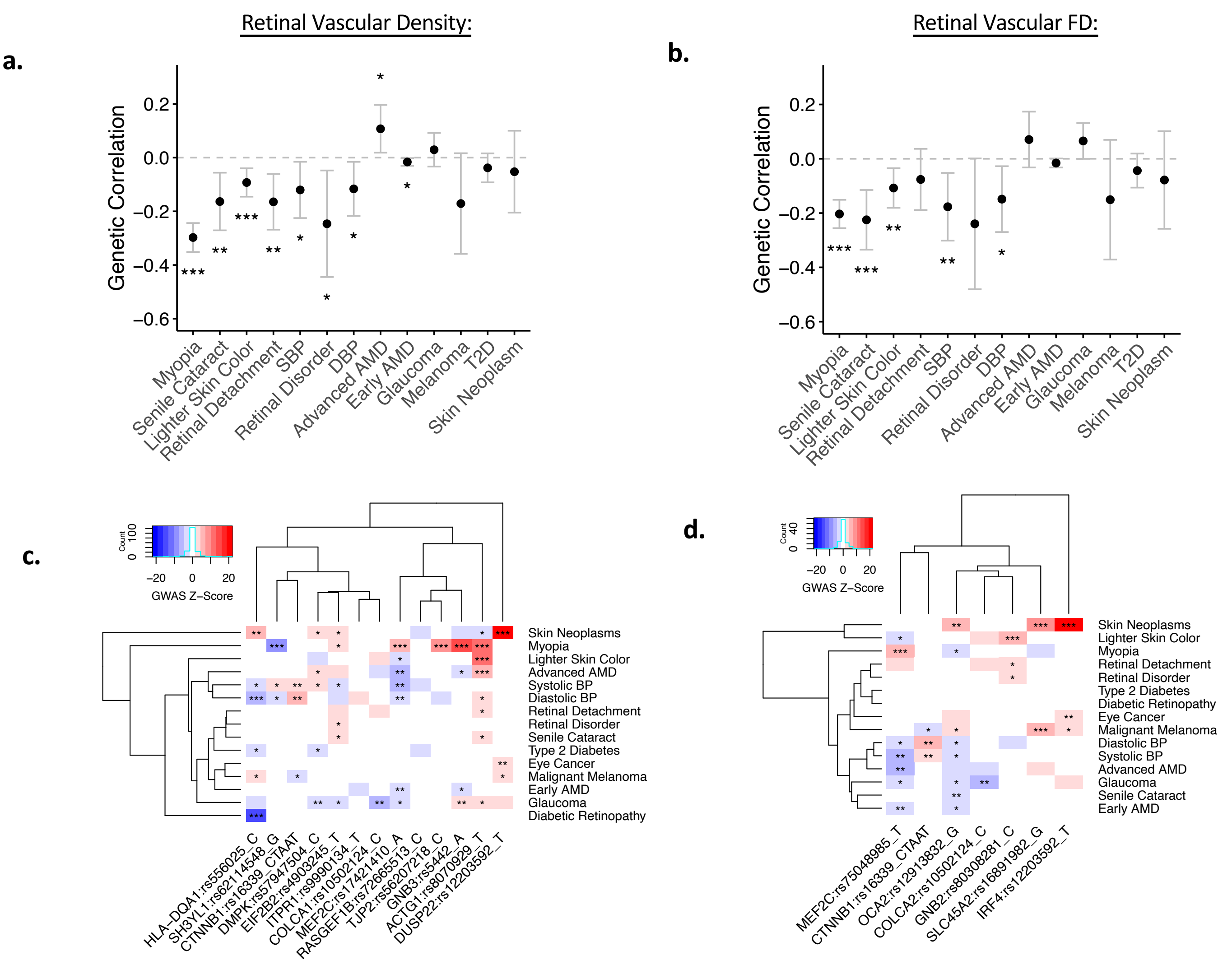


**Supplementary Figure 16: Genetic correlation analyses and fine-mapping for retinal vascular density and FD.** a,b) Genetic correlation analysis for vascular density and FD with other published GWAS studies (see legend of **Supplementary Table 13** for GWAS study details). For the genetic correlation analysis: ***:P<0.0001, **: 0.0001<P<0.01, *: 0.01<P<0.05. c,d) The 7 and 13 top finemapped variants at each PoPS-prioritized gene for FD and vascular density, and their association with other phenotypes. GWAS Z-scores reflect the GWAS z-score (ie: beta/se) for the respective GWAS outcome and the listed FD- or density- lowering allele. For the finemapping analysis, ***: P<5x10-8, **: 5x10$^{-8}$<P<0.0001, *: 0.05<P<0.0001. AMD = age-related macular degeneration; SBP = systolic blood pressure; DBP = diastolic blood pressure; FD = fractal dimension; GWAS = genome-wide association study; T2D = type 2 diabetes mellitus

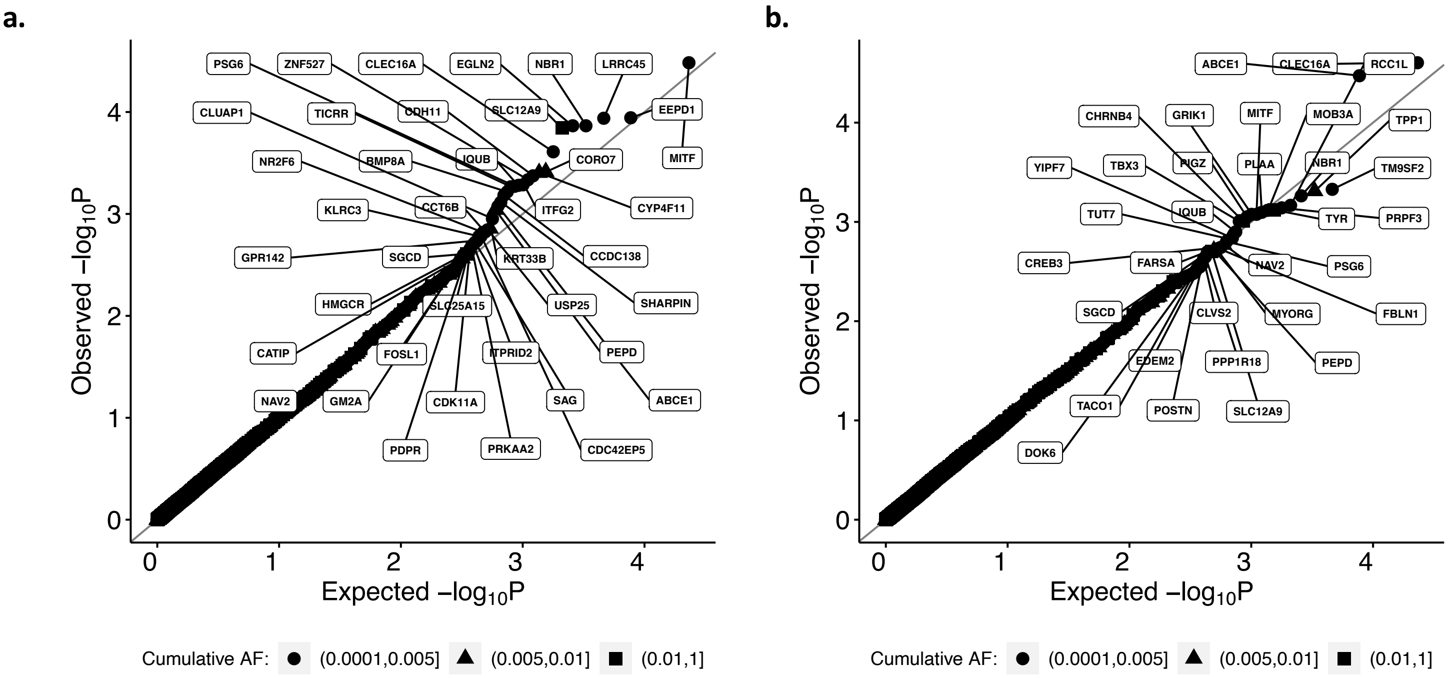


**Supplementary Figure 17: Rare variant association analysis of loss of function and missense variants grouped by gene for retinal vascular a) FD and b) density**. Associations are plotted stratified by cumulative allele frequency for each gene. AF = allele frequency; FD = Fractal dimension

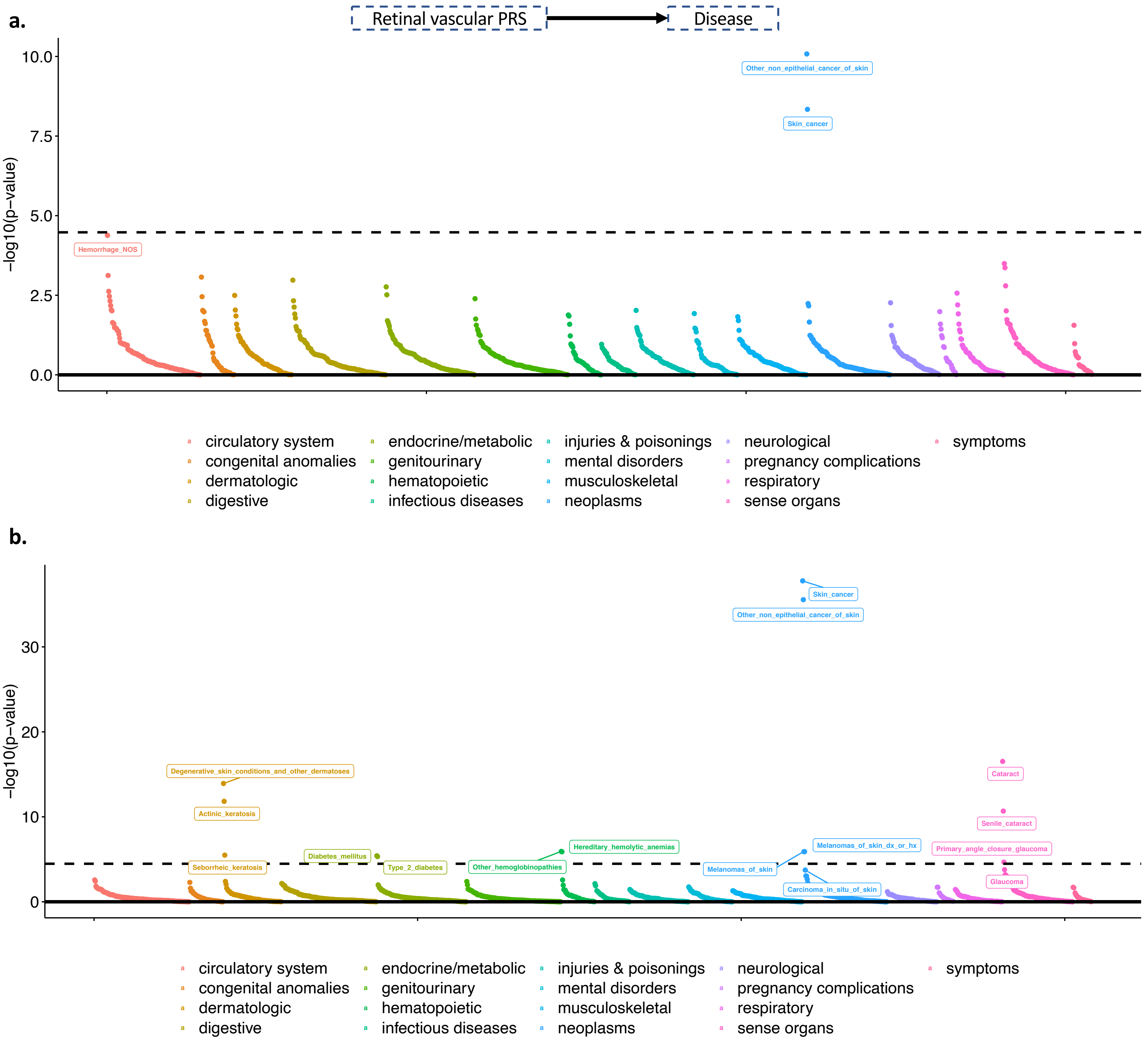


**Supplementary Figure 18: PheWAS of polygenic risk scores for a) vascular density and b) FD.** Horizontal dotted lines reflect the Bonferroni p-value for significance based on the number of analyzed phenotypes**.**

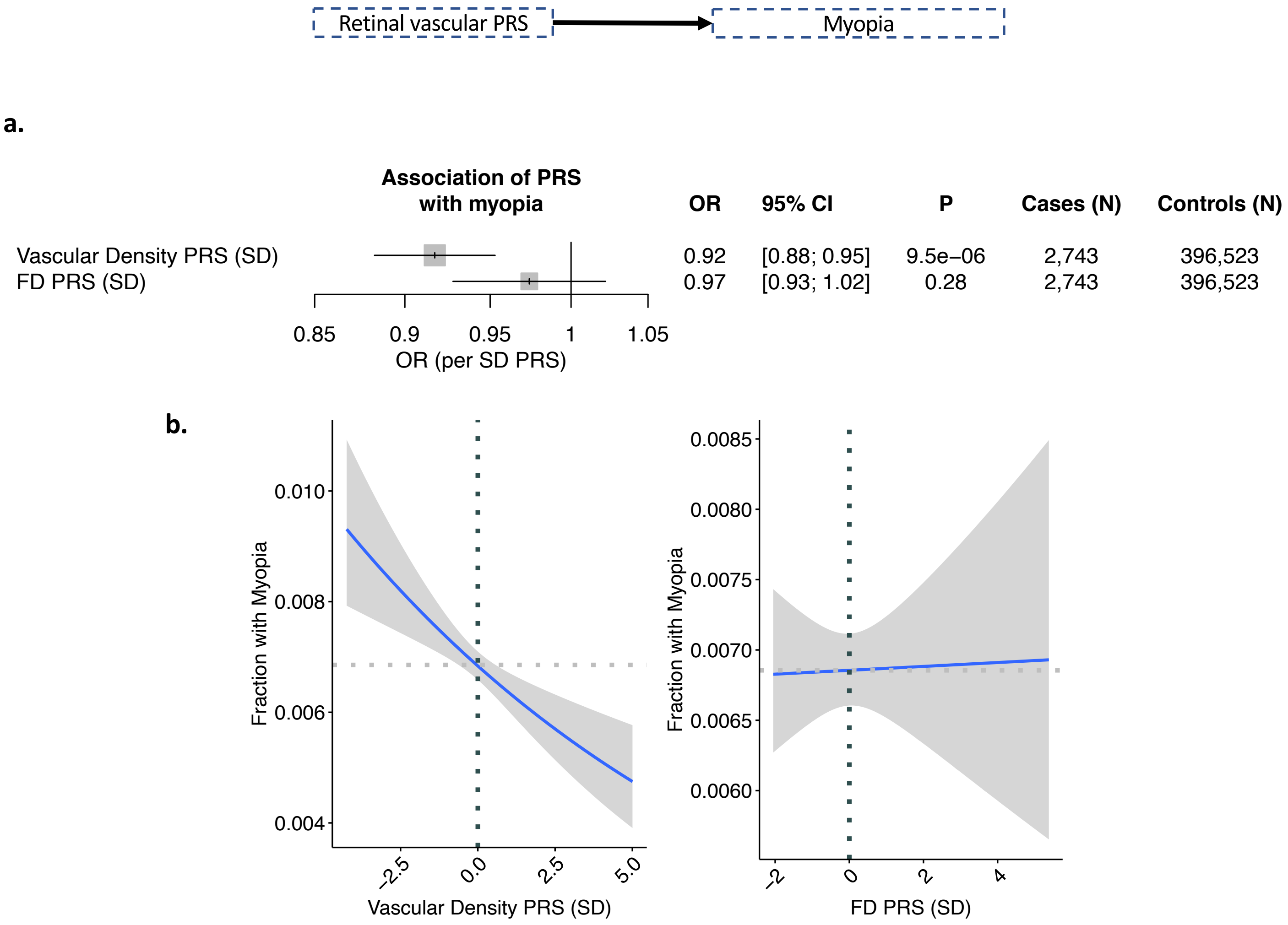


**Supplementary Figure 19: One-sample Mendelian Randomization for FD, vascular density PRS on myopia**. a. Association of the vascular density PRS and FD PRS with combined prevalent and incident myopia in a logistic regression model that includes the following covariates: age, age$^2$, sex, smoking status, and the first 10 principal components of genetic ancestry. b. Relationship of vascular density PRS and vascular FD PRS with fraction of individuals developing myopia during their lifetime. Horizontal and vertical dotted lines in panel b. reflect the median fraction with myopia (horizontal line) across the dataset, and the median vascular density PRS (vertical line at 0.0 SD), respectively. Shaded grey region reflects the 95% confidence interval using a restricted maximum likelihood (REML) binomial generalized additive model with integrated smoothness (from the gam() function in R).

FD = fractal dimension; PRS = polygenic risk score
